# Retrospective analysis of a dedicated care pathway for nonalcoholic fatty liver disease in an integrated US healthcare system demonstrates support of weight management and improved ALT

**DOI:** 10.1186/s12876-020-01492-9

**Published:** 2020-10-31

**Authors:** Heather Patton, Raoul Burchette, Stephanie Tovar, Jose Pio, Jiaxiao Shi, Lisa M. Nyberg

**Affiliations:** 1 Southern California Permanente Medical Group, Division of Gastroenterology, Garfield Specialty Center, 5893 Copley Drive, San Diego, CA 92111 USA; 2 Section of Gastroenterology, VA San Diego Healthcare System, 3350 La Jolla Village Drive, San Diego, CA 92161 USA; 3 Department of Research and Evaluation, Kaiser Permanente Research, Southern California Permanente Medical Group, 100 S. Los Robles Ave., Pasadena, CA 91101 USA

**Keywords:** Nonalcoholic fatty liver disease (NAFLD), Non-invasive fibrosis test, Clinical management, Weight loss

## Abstract

**Background:**

A care pathway for nonalcoholic fatty liver disease (NAFLD) in Kaiser Permanente San Diego, California was instituted in August 2017 to improve efficiency of disease staging and promote lifestyle modification.

**Methods:**

The NAFLD Care Pathway includes: (1) patient education (2) vibration controlled transient elastography (VCTE) examination (3) hepatology consultation for VCTE ≥ 8 kPa and (4) referral to weight management (WM). Patients referred to the pathway during the first 6 months of its implementation were studied for adherence to its components and impact on weight change and ALT values in the 12 months following referral. Retrospective assessment of WM participation, change in weight, and change in ALT were evaluated in the 12-months following referral and compared to changes 12-months prior. Student’s t-test or Wilcoxon signed rank test were used as appropriate (*p* < 0.05).

**Results:**

632 patients were included. 575 (91.0%) completed VCTE examination with mean liver stiffness 8.5 kPa (SD 9.2). 52 patients had mean liver stiffness ≥ 15 kPa. 180/632 (28.5%) attended NAFLD education. 153/632 (24.2%) were offered hepatology clinic and 136/153 (88.9%) completed at least 1 appointment. Participation in WM was 24/632 (3.8%) prior to referral and 67/632 (10.6%) after referral and increased among patients who attended NAFLD education. Mean weight change following referral was − 0.69 kg (SD 6.58 kg) among patients without WM and − 7.78 kg (SD 13.43 kg) with WM. Overall, 44.2% of participants experienced weight gain after referral, 40.8% had weight loss < 5% and 15% had weight loss ≥ 5%. Variables associated with weight loss included WM (*p* < 0.0001) and higher liver stiffness (*p* = 0.0066). Mean ALT change was − 15.2 (SD 38.5) U/L without WM and − 28.8 (SD 29.6) U/L with WM.

**Conclusions:**

A care pathway for NAFLD within a large, integrated healthcare system provides non-invasive disease staging and minimizes hepatology clinic utilization to those with more advanced disease. Referral was associated with increased enrollment in WM, weight loss, and decreased ALT. Given its impact on healthcare resources, strategies to improve NAFLD identification, staging, and promotion of lifestyle modification are imperative.

## Background

Nonalcoholic Fatty Liver Disease (NAFLD) is a highly prevalent condition commonly seen in association with metabolic comorbidities such as obesity, diabetes mellitus (DM), and dyslipidemia [1–3]. NAFLD encompasses a spectrum of severity ranging from typically non-progressive simple steatosis or nonalcoholic fatty liver (NAFL) to nonalcoholic steatohepatitis (NASH) characterized by inflammation, hepatocellular ballooning injury, and progressive fibrosis [2]. Distinguishing NAFL from NASH in clinical practice can be difficult but is relevant given that the majority of liver-related morbidity and mortality is seen in NASH and liver fibrosis is the most important variable predictive of prognosis [4]. For purposes of resource allocation, identification of patients with NASH serves to prioritize patients in greatest need of evaluation by gastroenterology and hepatology specialists [3].

Despite its high prevalence, there are significant knowledge gaps among primary providers in the diagnosis and management of NAFLD [5, 6]. The heavy clinical burden from NAFLD mandates partnership between primary care and specialty care with development of mutually agreeable triage processes to link high-risk patients with NAFLD to gastroenterology and hepatology providers. Optimal means of triage of NAFLD patients at the primary care setting should be performed with readily available clinical variables. Non-invasive testing, including both blood and imaging-based modalities, can be used for this purpose [7]. Additionally, the presence of Metabolic Syndrome (MetS) in NAFLD is associated with NASH and thus can help to target patients for additional assessment [8].

Kaiser Permanente (KP) is a large, integrated healthcare system with high penetrance in Southern California, serving approximately 650,000 members in San Diego. A dedicated care pathway for NAFLD in KP San Diego (KPSD) was implemented in 2017 with the goals of (1) assisting primary care providers (PCPs) in identifying patients with NAFLD most likely to have NASH and/or fibrosis (2) high throughput triage of NAFLD patients with noninvasive testing (3) provision of patient education regarding NAFLD diagnosis and (4) promotion of lifestyle modification programs through the healthcare plan aimed to promote weight loss. The primary aim of this study is to determine the initial success of this NAFLD care pathway in achieving these goals and the impact of this program on weight loss and improvement in liver blood tests.

## Methods

A dedicated care pathway for NAFLD was implemented at KPSD in August 2017. Components of the care pathway include:

- Education of primary care providers (PCPs) regarding patient populations at risk for NAFLD and NASH, clinical evaluation for NAFLD, and associated cardiovascular risks in this population. This education was administered as a didactic session during a mandatory primary care Continuing Medical Education meeting. High-risk NAFLD patients were defined for PCPs as those with body mass index (BMI) ≥ 30 kg/m^2^, age ≥ 50 years, and diabetes (DM).
- Automated approval of any Gastroenterology consultation placed with indication “Fatty Liver”
- All fatty liver referrals are offered vibration controlled transient elastography (VCTE, FibroScan®) examination, education and referral to KPSD weight management (WM). FibroScan® examinations were performed by trained operators in the Gastroenterology Department and results were reviewed and interpreted by hepatology physicians.
- NAFLD Education was developed by a hepatologist (HP) and delivered by trained health educators (registered nurses) in a 90-min classroom session in the Health Education Department. The curriculum includes a definition of NAFLD and its spectrum, tests used in the diagnosis and staging of NAFLD, risk factors, potential health outcomes, and data regarding the efficacy of lifestyle modification in improving or reversing NAFLD. Information about WM programs offered by the healthcare plan are also reviewed during the education session.
- Patients with liver stiffness measurement (LSM) ≥ 8 kPa are contacted and offered a clinic visit with Hepatology. The threshold of 8 kPa was selected as this would identify patients with an estimated liver fibrosis stage of 2 [9]. Patients with LSM < 8 kPa are informed that if they pursue lifestyle modification with modest weight reduction that fatty liver may be improved or reversed.

Primary Objectives:

- Improvement in liver blood tests (ALT) at 12 months following referral relative to change in ALT in the 12 months prior to referral (historical period).
- Change in weight at 12 months following referral relative to change in weight in the historical period.
  Secondary Objectives:
- Enrollment in WM programs through KPSD.
- Performance of VCTE (FibroScan®) as a triage measure for NAFLD (rate of exam completion, technical success for exams performed, and percentage patients identified with advanced liver fibrosis).
- Patient participation in NAFLD education.
- Utilization of hepatology clinic for NAFLD patients identified with LSM ≥ 8 kPa.
- Change in HgbA1C at 12 months following referral relative to change in HgbA1C in the historical period.
- Determination of factors associated with weight loss and change in ALT.

### Design

This is a retrospective analysis of patients referred to the KPSD NAFLD Care Pathway during the first 6 months of its implementation. This study was approved by the KP Southern California (KPSC) Institutional Review Board prior to initiation and patients were de-identified for analysis. Patients were identified for study through a clinical database maintained by hepatology case managers and included after verification that their referral occurred between August 1, 2017 and January 31, 2018 with an International Classification of Diseases (ICD) Code for NAFLD/NASH (K76.0, K75.81). Patients were excluded from study if there was evidence of Gastroenterology referral or specialty care outside of the study window, a gap in healthcare coverage exceeding 45 days during the study period, or an ICD code indicating an alcohol use disorder or alcoholic liver disease (K70.0, K70.30, K70.9, K77.xx, F10.xx). For purposes of evaluating study end points, patient outcomes were assessed at 12 months following their referral date and compared to historical patient data assessed at 12 months prior to their referral date.

Baseline patient characteristics were determined at the time of the referral and included demographics, anthropometrics, comorbid medical conditions, and laboratory data.

Diabetes/prediabetes and hypertension (HTN) were determined from Southern California KP disease registries. KP disease registries require a combination of ICD codes (2 or more), laboratory results (for diabetes) plus pharmacy dispensing records to identify patients. Dyslipidemia was identified through ICD codes (E78.00, E87.01, E78.1, E78.2, E78.5). Cardiovascular disease was defined according to a comprehensive set of ICD codes (available as supplemental data in Additional file 1). VCTE (FibroScan®) reports were evaluated for date of exam, median liver stiffness measurement (LSM) and interquartile range % (IQR%). VCTE exams with IQR% > 30 were deemed invalid and excluded from analysis. Hepatology utilization was assessed by documentation of any hepatology visit type (telephone or clinic) following referral among patients with LSM ≥ 8 kPa. Chart review was utilized to determine attendance at NAFLD class. Participation in WM programs included attendance at any of the programs offered through KPSD during the historical and study periods, including full and partial fasting programs (OPTIFAST®) and Healthy Balance (16 weekly group sessions promoting plant-based nutrition and physical exercise).

NAFLD Fibrosis score (NFS) was calculated from data, where available, within 6 months of referral from the following formula [10]:

$$\begin{aligned}{\text{NAFLD fibrosis score}} & = - 1.675 + 0.037 \times {\text{age}}\,({\text{years}}) + 0.094 \times {\text{BMI}}\,({\text{kg/m}}^{2}) \\& \quad + 1.13 \times {\text{impaired fasting glucose/diabetes}}\,({\text{yes}} = 1,\,{\text{no}} = 0) \\& \quad + 0.99\times {\text{AST}}/{\text{ALT ratio}} - 0.013 \times {\text{platelet}}\,(\times 10^{9} /{\text{L}}) \\ & \quad - 0.66 \times {\text{albumin}}\,( {\text{g}}/{\text{dL}}). \end{aligned}$$

NFS was categorized as low risk (< − 1.455, high negative predictive value for advanced fibrosis), indeterminate risk (− 1.455 to 0.676) or high risk for advanced liver fibrosis (> 0.676, high positive predictive value for advanced fibrosis). Weight change following referral to the NAFLD Care Pathway was categorized as: weight gain (any), < 5% weight loss, 5–10% weight loss or ≥ 10% weight loss. Change in ALT was assessed for each of these categories for those patients who had ALT data in the post intervention study period.

### Statistical analysis

Change in body weight (kilograms), change in ALT (U/L), and change in HgbA1c (%) were calculated during the historical period (from data closest to 12 months prior to referral to time of referral) and compared to changes in these variables during the study period (from time of referral to data available closest to 12 months from referral). Student’s t-test or Wilcoxon signed rank test were used as appropriate (significance set at *p* < 0.05).

Linear regression was performed to evaluate factors associated with weight loss and ALT improvement in the study population. Variables assessed included attendance at NAFLD education, having had a hepatology clinic visit, enrollment in weight management programs, LSM results, presence of comorbidities (DM, HTN, and dyslipidemia), and whether patients met definition for high risk NAFLD (DM + age ≥ 50 years + BMI ≥ 30 kg/m^2^). The covariates were examined by correlation for multi-colinearity and other native associations. Ad hoc models of change in outcome between periods were built using both stepwise selection and backward elimination to verify that the model was reasonably stable if both methods produced the same results using successively stricter thresholds for entry and removal (both at 0.15 and then both at 0.05). We included covariates that were close to the final threshold.

## Results

**Fig. 1:**
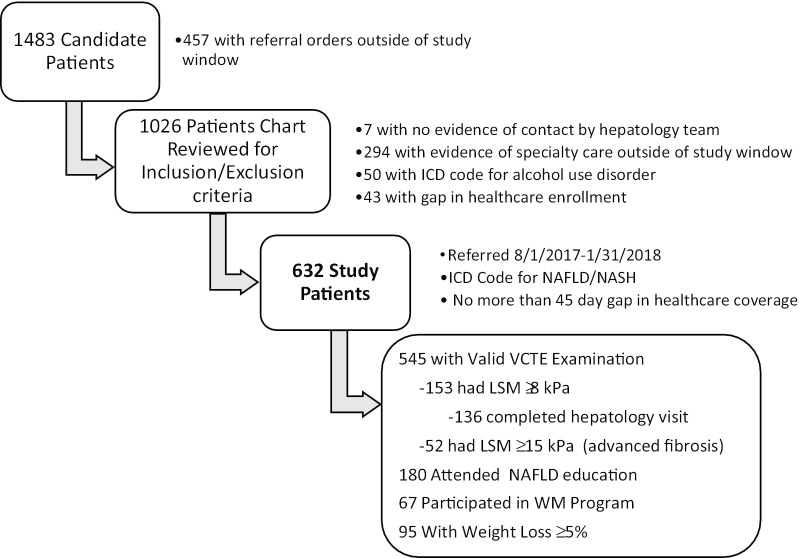
Flow Chart of patients referred to NAFLD Care Pathway to arrive at study population

A total of 632 patients referred to the NAFLD Care Pathway during the initial 6 months of its implementation (August 1, 2017–January 31, 2018) met inclusion and exclusion criteria for study (Fig. 1). Patient characteristics are summarized in Table 1. At baseline (time of referral), the mean age was 53.7 years with female predominance (56.7% vs. 43.4% males). White race was most prevalent, followed by Hispanic, Asian and Black. Obesity prevalence was high, with a mean BMI of 34 kg/m^2^. Comorbidities included in the Metabolic Syndrome were common (diabetes/prediabetes: 32.6%, dyslipidemia: 59.7%, and hypertension: 48.3%). Nearly 10% of the population had a diagnostic code for cardiovascular disease. Liver biochemistries were mildly abnormal (mean ALT 59.4 U/L, AST 41.8 U/L, GGT 78.3 U/L) while baseline albumin, INR and bilirubin were within normal range (3.9 g/dL, 1.0 and 0.7 mg/dL, respectively). Baseline platelet count was normal at 240.1 (× 10^9^/L).

**Table 1:** Baseline characteristics of patients referred to the NAFLD care pathway 8/1/2017–1/31/2018

Population characteristic	Total
Study population referred to NAFLD Care Pathway 8/1/2017–1/31/2018	632
Gender, N (%)	
Male	274 (43.4%)
Female	358 (56.7%)
Mean age (SD) years	53.7 (13.0)
Race/ethnicity, N (%)	
White	271 (42.9%)
Hispanic	247 (39.1%)
Asian	66 (10.4)
Black	20 (3.2%)
Other/unknown/multiple	28 (4.4%)
Mean BMI (SD) kg/m^2^	34.0 (6.8)
Diabetes/Prediabetes, N (%)	206 (32.6%)
Dyslipidemia, N (%)	377 (59.7%)
HTN, N (%)	305 (48.3%)
Cardiovascular Disease, N (%)	62 (9.8%)
ALT U/L, mean (SD) (N = 602)	59.4 (42.9)
AST U/L, mean (SD) (N = 405)	41.8 (36.4)
GGT U/L, mean (SD) (N = 384)	78.3 (104.2)
Albumin g/dL, mean (SD) (N = 198)	3.9 (0.3)
Total Bilirubin mg/dL, mean (SD) (N = 520)	0.7 (0.4)
Platelet count (× 10^9^/L) mean (SD) (N = 517)	240.1 (72.0)
INR, mean (SD) (N = 171)	1.0 (0.3)
Ferritin ng/mL, mean (SD) (N = 228)	239.9 (255.5)
HgbA1c %, mean (SD) (N = 556)	6.3 (1.2)
Total Cholesterol mg/dL, mean (SD) (N = 479)	182.7 (44.6)
LDL mg/dL, mean (SD) (N = 467)	107.2 (36.9)
HDL mg/dL, mean (SD) (N = 479)	45.4 (11.5)
Triglycerides mg/dL, mean (SD) (N = 329)	176.8 (253.8)

Data shown for the initial cohort of patients referred to the NAFLD Care Pathway upon its creation in August 2017. All data shown are from the time of referral. Laboratory values of interest were not available in all participants in this retrospective analysis and thus numbers for which individual results are available are shown in parentheses. Comorbid health conditions were identified through ICD codes and KP disease registries

Flow chart illustrating disposition of candidate patients initially identified through a clinical database maintained by case managers in hepatology. Candidate patients were restricted to those referred during the initial 6 months of the care pathway’s implementation and then further subjected to exclusion criteria. A total of 632 patients were included in this analysis and adherence to aspects of the care pathway are shown.

### High-risk NAFLD

**Fig. 2:**
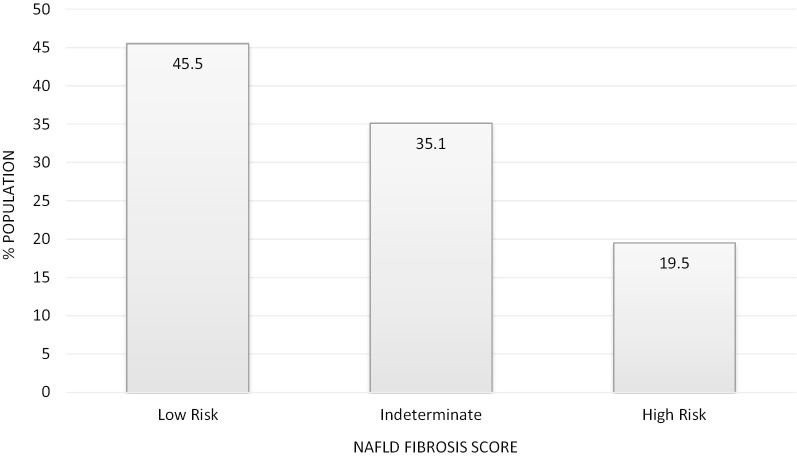
NAFLD Fibrosis score results at time of referral

A total of 101 (16%) patients met pre-defined high-risk NAFLD criteria (age ≥ 50 years, DM, and BMI ≥ 30 kg/m^2^). High-risk NAFLD patients (n = 40 with available labs) had mean ALT of 55.1 U/L (range 14–111, SD = 21.6) vs. 66.5 U/L (range 10–287, SD = 43.8) for the remaining NAFLD patients (n = 179 with available labs) (*p* = 0.295 [based on chi-square for Wilcoxon rank-sum statistic]). Data were available to calculate a NAFLD Fibrosis Score in 154/632, most of whom (80.6%) were in the low or indeterminate risk categories (Fig. 2).

Data shown for 154 patients with available labs to calculate a NAFLD Fibrosis Score at the time of referral. Results of the NAFLD Fibrosis Score are categorized as low risk (< − 1.455), indeterminate (− 1.455 to 0.675), and high risk (> 0.675) and reported as percent of the 154 with available data within each category.

### VCTE results

**Fig. 3:**
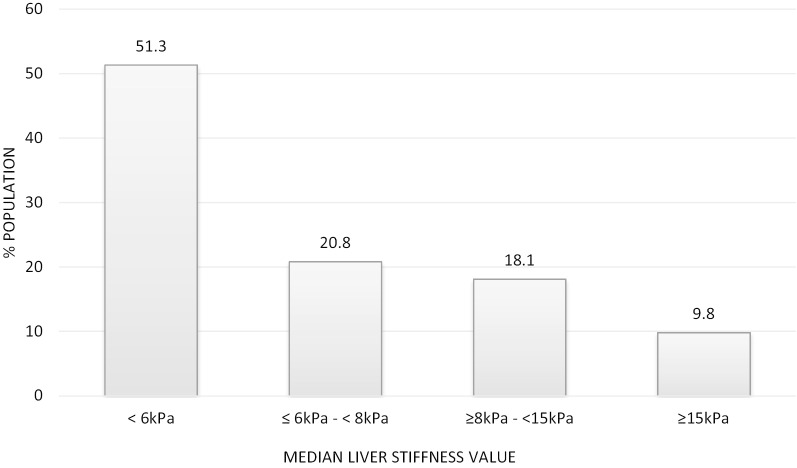
Breakdown of liver stiffness measurements on VCTE examination

575 (91.0%) patients completed VCTE (FibroScan®) examination and 545 (94.8%) exams were deemed reliable (IQR ≤ 30%). Mean LSM results for the entire study population was 8.5 kPa (SD 9.2). Most patients (286/545 = 52.5%) had normal liver stiffness measurement (< 6 kPa) (Fig. 3). 392 patients (71.9%) had LSM < 8 kPa and thus were not offered an appointment in hepatology while 153 patients (28.1%) had LSM results ≥ 8 kPa and were offered an appointment in hepatology clinic. 52 patients (9.5%) had LSM ≥ 15 kPa indicating possible advanced liver fibrosis/cirrhosis. Median LSM was 7.4 kPa (range 2.2–70.7, SD = 11.84) for high-risk NAFLD vs. 5.6 kPa (range 2.2–75.0, SD = 8.93) for the remaining NAFLD patients (*p* < 0.001 [based on chi-square for Wilcoxon rank-sum statistic]).

Results of 545 VCTE (FibroScan®) examinations performed in 575 patients referred to the NAFLD Care Pathway with IQR ≤ 30%. Data shown are for the percent of these 545 examinations with median liver stiffness measurement in categories of increasing severity.

### NAFLD education

A total of 180/632 (28.5%) patients attended the single NAFLD education session that was offered to all patients.

### Hepatology clinic utilization

A total of 153/632 (24.2%) patients were offered a hepatology clinic visit based on their VCTE examination results (LSM ≥ 8 kPa). 136 of 153 (88.9%) patients offered appointments completed at least 1 hepatology clinic visit within 1 year of referral (representing 21.5% of the study population). There were a total of 188 visits completed by the 136 patients with 25 patients completing at least 1 telephone visit and 126 patients completing at least 1 office visit.

### WM program

Participation in a KP WM program was 24/632 (3.8%) in the 12 months prior to referral and 67/632 (10.6%) after referral. Participation in WM after referral was greater (14.4%) among those who attended NAFLD Class versus those who did not (9.1%) (*p* = 0.048) (Table 2). There was no association between NAFLD class attendance and participation in WM programs prior to referral to the NAFLD Care Pathway (*p* = 0.70).

**Table 2:** Relationship between weight management program and NAFLD class participation

NAFLD class attendance	Weight management program participation following referral		%
	No	Yes	
No	411	41	9.1
Yes	154	26	14.4

Patient participation in any of the KP Weight Management Programs in the 12 months following referral to the NAFLD Care Pathway shown according to whether patients attended the single NAFLD Education session offered to all patients. Weight management program participation was higher in the subset of patients who had attended NAFLD Class (*p* = 0.048).

### Change in weight

**Fig. 4:**
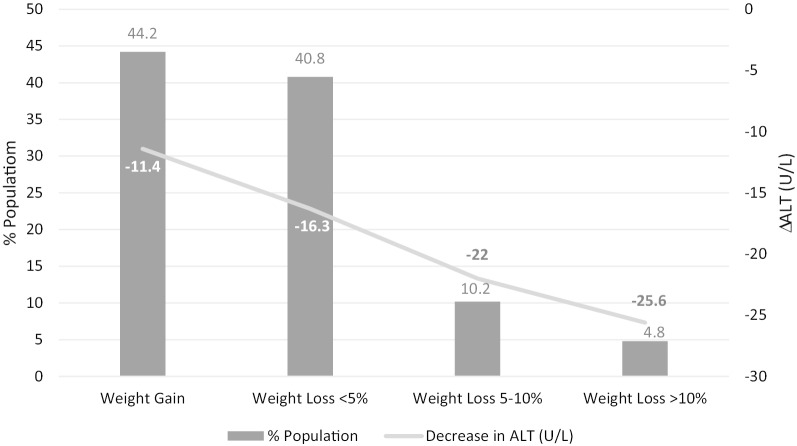
Change in ALT value according to change in weight following referral to the NAFLD care pathway

In the 12 months prior to referral, mean weight change was + 0.09 kg (SD 4.85 kg). Mean weight change following referral was − 1.45 kg (SD 7.94 kg) overall, − 0.68 kg (SD 6.58 kg) among patients who did not enroll in WM programs and − 7.80 kg (SD 13.43 kg) with WM program participation. While over 50% of patients achieved some weight loss following referral, only a minority of patients achieved weight loss in excess of 5% (Fig. 4). Change in mean ALT (standard deviation) was determined according to weight change category in the post referral period: − 11.4 U/L (35.7) in 83 patients with weight gain (any), − 16.3 U/L (27.1) in 72 patients with < 5% weight loss, − 22.0 U/L (40.3) in 28 patients with 5–10% weight loss, and − 25.6 U/L (39.3) in 25 patients with ≥ 10% weight loss (Fig. 4). On linear regression analysis, the variables associated with weight loss after referral included participation in WM program (*p* < 0.0001) and higher LSM result on VCTE examination (*p* = 0.007).

The bar graph depicts percent of patients in each category of weight change among 632 patients referred to the NAFLD Care Pathway in the 12 months following the referral date. Overlying this, the line graft depicts the mean change in ALT [ΔALT = (ALT value 12 months after referral) − (ALT value at time of referral)] for patients within each weight change category. As percentage weight loss increased, the average decline in ALT value during this time interval was also increased.

### Change in ALT

The average change in ALT among those who had values in both periods (n = 219) was − 3.1 U/L (SD 43.6) prior to referral and − 15.4 U/L (SD 35.2) following referral. Mean ALT change over the entire study period was − 17.1 (SD 38.1) U/L overall, − 15.2 (SD 38.5) U/L without WM and − 28.8 (SD 29.6) U/L with WM. On linear regression analysis, no significant predictors of decrease in ALT were identified aside from higher baseline ALT value.

Change in ALT after the index date (following referral to the care pathway) based on NAFLD class attendance and WM participation was evaluated in the 219 patients with ALT results available throughout the study period. The 82 patients who took NAFLD Class had a reduction in ALT during the follow-up period of 17.1 U/L compared to a reduction in ALT of 14.5 U/L in the 137 patients who did not take NAFLD class (*p* = 0.11, Wilcoxon rank sum comparison). Change in ALT in the follow-up period among the 38 patients who enrolled in WM was a decrease of 25.7 U/L compared to a decrease of 13.3 U/L in the 181 patients who did not enroll in WM (*p* = 0.03 for the Wilcoxon rank sum comparison).

### Change in HgbA1c

A total of 310 patients had HgbA1c results available in both the pre- and post-referral periods. The change in HgbA1c among those who had values in both periods was a decrease from an average of 6.5% before referral to an average of 6.2% after referral (*p* = NS).

## Discussion

Given the high prevalence of NAFLD with estimated burden in the US approaching 100 million, specialty evaluation of all patients is untenable [11]. The optimal setting, modality, and timing of triage for specialty referral remains to be determined (and may vary according to healthcare system, driven by factors such as patient population characteristics and available resources). We have demonstrated the feasibility and clinical benefits of implementation of a dedicated care pathway for NAFLD within a large, integrated healthcare system in the US. Our evaluation of over 600 patients referred in the initial six months of this program suggests that primary care providers will utilize available resources for their patients with NAFLD, that patients with NAFLD are willing to pursue education and additional noninvasive testing (NIT) for their diagnosis, that patient education increases enrollment in weight management programs, and that weight management programs improve weight reduction and decrease in ALT.

The risk for liver-related mortality in NAFLD increases exponentially with stage of liver fibrosis, highlighting the importance of fibrosis assessment in clinical practice [4]. The American Association for the Study of Liver Disease (AASLD) Practice Guidelines support use of NIT for evaluating patients with NAFLD and to help guide identification of patients who may be appropriate for liver biopsy [2]. NAFLD Fibrosis Score and FIB4 both perform well in identifying patients with NAFLD who have advanced liver fibrosis [12]. Furthermore, high NFS score has been identified as a marker of increased mortality (pooled RR 4.54, 95% CI 1.85–11.17) thus either or both of these readily available and inexpensive scoring systems are helpful tools in staging and prognosis of patients with NAFLD [13]. Calculation of NAFLD Fibrosis Score or FIB4 were not included in the initial KPSD NAFLD Care Pathway algorithm, but all patients were offered staging through VCTE. Patient adherence to this examination was excellent (91%) and this approach identified 153 patients with increased likelihood of significant liver fibrosis, including 53 with LSM ≥ 15 kPa indicating high positive predictive value for cirrhosis [9]. Higher LSM in this study population was associated with weight loss subsequent to referral. Potential explanations for this observation include loss of fat and muscle stores in patients with advanced liver disease or the possibility that the communication of disease staging results may help to motivate patients in adopting lifestyle modification.

The NAFLD care pathway developed for KPSD minimized utilization of subspecialty providers to 152/632 (24.1%) of patients with potentially advanced disease thus having a favorable impact on access to hepatology care. Improved recognition of NAFLD populations at risk for advanced disease may help to identify patients in the pre-cirrhotic and early cirrhotic stages when effective therapy may help to halt or reverse disease progression and associated healthcare burdens. NAFLD may not be recognized or addressed prior to development of overt cirrhosis, often identified as an incidental finding in which case concomitant hepatocellular carcinoma (HCC) may be present [14]. In fact, NAFLD is emerging as the most common etiology of HCC [15, 16]. Early identification of NAFLD patients with advanced fibrosis provides opportunity to implement HCC surveillance examinations, addressing data in the US that indicate HCC screening is performed less often in NAFLD cirrhosis and the diagnoses of HCC in NAFLD-related liver disease tends to occur later with more advanced cancers than other liver disease etiologies [17–19].

Our results demonstrate that PCPs are not intuitively referring high risk NAFLD patients for care as demonstrated by the low percentage of patients who met pre-defined high-risk criteria (16%) and high risk for advanced fibrosis by NAFLD Fibrosis Score (19%). As specialists, we need to partner with referring providers to ensure that they have the appropriate knowledge of NAFLD patients in high risk categories, are comfortable with clinical tools for risk assessment such as FIB4 and NAFLD Fibrosis Score and have access to resources to support patients in their efforts at lifestyle modification [6]. In KP Southern California, the electronic medical record (EMR) now has an embedded NAFLD Fibrosis Score calculator that conveys results with interpretation. Such automated tools, particularly when incorporated into the EMR, offer the best opportunity to support overburdened practitioners in assessing NAFLD. Primary care-based assessment with NIT, including laboratory-based scores and VCTE, has been shown to be cost effective and to markedly reduce referral for specialty care [20, 21]. Future iterations of this KPSD NAFLD Care Pathway should incorporate NIT at the level of the referring provider to further optimize resource utilization and improve the financial sustainability of this program.

While there are several pharmacotherapies under investigation for the treatment of NASH, lifestyle modification remains the standard of care for management of NAFLD [2, 22]. Nutrition, exercise, and weight loss are effective at improving NASH but are difficult to institute and sustain in clinical practice [23, 24]. Exercise alone or combined with dietary intervention, even absent weight loss, may reduce intrahepatic fat content [25]. A 5–10% weight loss via dietary intervention in combination with exercise is recommended for management of NAFLD yet resources to support these behaviors are rarely available outside of the context of a clinical trial and success is likely hampered by a lack of clinical training in effective behavior change interventions [24, 26]. Data from the National Health and Nutrition Examination Survey (NHANES) indicate that physician counseling for weight loss among patients with NAFLD occurs at low rates (46%). While this counseling is associated with patient reported effort to lose weight, it was not demonstrated to improve rates of significant (≥ 5%) weight reduction [27]. Our real-world experience in a large-scale program to encourage lifestyle modification for NAFLD demonstrated a nearly threefold increase in WM program participation (10.6% versus 3.8%) with greater enrollment in WM programs among those who had attended NAFLD education. Not surprisingly, participation in WM programs was associated with greater weight loss, as was a higher LSM result on VCTE exam. Furthermore, 10% of patients referred to this care pathway achieved a weight loss in the 5–10% range and nearly 5% of patients achieved a weight loss ≥ 10%.

This study has several important limitations to acknowledge. As a retrospective analysis, variables of interest were not available for the entire study population at all time points of interest and ICD codes were used for the exclusion of alcohol related liver disease. While we were able to capture enrollment in WM programs offered through the healthcare plan, we were not able to account for participation in other formal or self-directed weight loss programs. Likewise, exercise is an important aspect of lifestyle modification for NAFLD with data supporting improved liver fat content, particularly when accompanied by weight loss [28]. While the benefits of exercise in management of NAFLD were included in NAFLD patient education, there was no routine assessment for physical activity level to determine if this parameter was impacted by the intervention. We also acknowledge that the duration of follow-up was relatively short such that the full benefits of the program as well as the durability of the intervention were not able to be assessed (both key in evaluating the sustainability of this intervention). Finally, these results may not be generalizable to healthcare systems that are not fully integrated and/or do not have internal resources such as weight management programs and a health education department to support this type of care delivery. The nature of healthcare in the US (lack of national healthcare or universal insurance coverage) results in tremendous heterogeneity in the approach to patient care for conditions like NAFLD and discrepancy in access to resources for weight management. Nearly 10% of national healthcare expenditure in the US is spent on prescription drugs, thus healthcare delivery systems may see favorable economic outcomes from the successful implementation of lifestyle modification programs in offsetting pharmaceutical utilization. Furthermore, particularly as therapies for NASH progress, payors may be compelled to support NIT programs for NAFLD to improve earlier identification of patients and provide opportunity to disrupt progression to advanced liver disease and its associated clinical and economic burdens [29].

Strengths of our study include our large patient population with greater than 600 individuals meeting study inclusion in the first six months of program implementation.

Despite the retrospective nature of this study, the large study population and robust tools available within this integrated healthcare system facilitated characterization of the study population. This study presents a unique, real world experience with implementation of systems to promote non-invasive testing, patient education/motivation, and lifestyle modification that offers both proof of concept as well as important lessons to modify future care pathways to optimal effect. While few patients achieved weight loss in excess of 5%, the fact that > 50% of patients lost some weight in the year following referral is encouraging. The ALT value decreased over the course of the study period and the decline in ALT was greater in those with weight reduction. We also captured very high adherence to disease staging with VCTE examination, identifying nearly 10% of the study population with potentially advanced liver fibrosis/cirrhosis.

## Conclusions

In conclusion, we have demonstrated the feasibility of implementing a dedicated care pathway for patients with NAFLD within a large, integrated healthcare system in the United States. This program was able to identify patients with liver fibrosis though VCTE examination and, in so doing, limit the impact of NAFLD on hepatology clinic utilization to those with evidence of advanced disease. Aided by patient education offered outside of the context of a clinic visit, this care pathway was successful in increasing enrollment in WM programs with over half of the patients achieving some weight loss in the 12 months following their referral. Liver blood tests (ALT) improved over the course of the study period, particularly among those with weight loss. Given the high prevalence of NAFLD, we believe that the implementation of care pathways within healthcare systems offer the opportunity to link patients with advanced fibrosis to specialty care prior to the onset of overt cirrhosis and to improve adherence to lifestyle modification.

## Supplementary information

**Additional file 1**. Comprehensive list of ICD9 and ICD 10 Codes used to identify atherosclerotic cardiovascular disease in study population as a comorbid health condition.

## Data Availability

Anonymized data that support the findings of this study are made available from the corresponding author upon reasonable request from qualified researchers with documented evidence of training in human subject protections.

## Abbreviations

NAFLD: Nonalcoholic fatty liver disease
KP: Kaiser Permanente
VCTE: Vibration controlled transient elastography
WM: Weight management
NAFL: Nonalcoholic fatty liver
NASH: Nonalcoholic steatohepatitis
MetS: Metabolic Syndrome
KPSD: KP San Diego
PCPs: Primary care providers
BMI: Body mass index
DM: Diabetes mellitus
LSM: Liver stiffness measurement
ICD: International Classification of Diseases
HTN: Hypertension
IQR%: Interquartile range %
NFS: NAFLD fibrosis score
NIT: Noninvasive testing
AASLD: American Association for the Study of Liver Disease
HCC: Hepatocellular carcinoma
EMR: Electronic medical record
NHANES: National Health and Nutrition Examination Survey
