# Supplementary material for: Retrospective analysis of a dedicated care pathway for nonalcoholic fatty liver disease in an integrated US healthcare system demonstrates support of weight management and improved ALT
Source: BMC Gastroenterol. 2020 Oct 31;20:362. doi: 10.1186/s12876-020-01492-9 (PMC7603663; doi:10.1186/s12876-020-01492-9)
Supplement: Supplementary file 1 — Additional file 1. Comprehensive list of ICD9 and ICD 10 Codes used to identify atherosclerotic cardiovascular disease in study population as a comorbid health condition. [file 12876_2020_1492_MOESM1_ESM.docx]

**Additional File 1:** Comprehensive list of ICD9 and ICD 10 Codes used to identify atherosclerotic cardiovascular disease in study population as a comorbid health condition.

**Summary of ICD9 & ICD10 Codes**

410.x1 Acute myocardial infarction, initial episode of care

411.xx Other acute and subacute forms of ischemic heart disease

413.xx Angina pectoris (excluding 413.1 – prinzmetal angina)

414.xx Other forms of chronic ischemic heart disease (excluding 414.1 – aneurysm and dissection of heart

433.xx Occlusion and stenosis of precerebral arteries

440.1 Atherosclerosis of renal artery

440.2x Atherosclerosis of native arteries of the extremities

440.3x Atherosclerosis of bypass graft of the extremities

440.4 Chronic total occlusion artery extremities

445.xx Atheroembolism

I20.x Angina pectoris, excluding I20.1 angina pectoris with documented spasm

I21.xx ST elevation and non-ST elevation myocardial infarction

I22.x Subsequent ST elevation and non-ST elevation myocardial infarction

I24.x Other acute ischemic heart disease, excluding I24.1 Dressler’s syndrome

I25.1xx Atherosclerotic heart disease of native coronary artery

I25.5 Ischemic cardiomyopathy

I25.6 Silent myocardial ischemia

I25.7xx Atherosclerosis of coronary artery bypass graft(s) and coronary artery of transplanted heart with angina pectoris

I25.8xx Other forms of chronic ischemic heart disease

I25.9 Chronic ischemic heart disease, unspecified

I63.2xx Cerebral infarction due to unspecified occlusion or stenosis of precerebral arteries

I63.5xx Cerebral infarction due to unspecified occlusion or stenosis of cerebral arteries

I65.xx Occlusion and stenosis of precerebral arteries, not resulting in cerebral infarction

I66.xx Occlusion and stenosis of cerebral arteries, not resulting in cerebral infarction

I67.2 Cerebral atherosclerosis

I70.xx Atherosclerosis, excluding I70.0, I70.8, I70.90 and I70.91

I75.xxx Atheroembolism

**Crosswalk ICD9 to ICD10 Codes**

| 410.01 | Acute myocardial infarction of anterolateral wall, initial episode of care | I2109 | ST elevation (STEMI) myocardial infarction involving other coronary artery of anterior wall |
| --- | --- | --- | --- |
| 410.01 | Acute myocardial infarction of anterolateral wall, initial episode of care | I220 | Subsequent ST elevation (STEMI) myocardial infarction of anterior wall |
| 410.11 | Acute myocardial infarction of other anterior wall, initial episode of care | I2101 | ST elevation (STEMI) myocardial infarction involving left main coronary artery |
| 410.11 | Acute myocardial infarction of other anterior wall, initial episode of care | I2102 | ST elevation (STEMI) myocardial infarction involving left anterior descending coronary artery |
| 410.11 | Acute myocardial infarction of other anterior wall, initial episode of care | I2109 | ST elevation (STEMI) myocardial infarction involving other coronary artery of anterior wall |
| 410.11 | Acute myocardial infarction of other anterior wall, initial episode of care | I220 | Subsequent ST elevation (STEMI) myocardial infarction of anterior wall |
| 410.21 | Acute myocardial infarction of inferolateral wall, initial episode of care | I2119 | ST elevation (STEMI) myocardial infarction involving other coronary artery of inferior wall |
| 410.21 | Acute myocardial infarction of inferolateral wall, initial episode of care | I221 | Subsequent ST elevation (STEMI) myocardial infarction of inferior wall |
| 410.31 | Acute myocardial infarction of inferoposterior wall, initial episode of care | I2111 | ST elevation (STEMI) myocardial infarction involving right coronary artery |
| 410.31 | Acute myocardial infarction of inferoposterior wall, initial episode of care | I221 | Subsequent ST elevation (STEMI) myocardial infarction of inferior wall |
| 410.41 | Acute myocardial infarction of other inferior wall, initial episode of care | I2119 | ST elevation (STEMI) myocardial infarction involving other coronary artery of inferior wall |
| 410.41 | Acute myocardial infarction of other inferior wall, initial episode of care | I221 | Subsequent ST elevation (STEMI) myocardial infarction of inferior wall |
| 410.51 | Acute myocardial infarction of other lateral wall, initial episode of care | I2129 | ST elevation (STEMI) myocardial infarction involving other sites |
| 410.51 | Acute myocardial infarction of other lateral wall, initial episode of care | I228 | Subsequent ST elevation (STEMI) myocardial infarction of other sites |
| 410.61 | True posterior wall infarction, initial episode of care | I2129 | ST elevation (STEMI) myocardial infarction involving other sites |
| 410.61 | True posterior wall infarction, initial episode of care | I228 | Subsequent ST elevation (STEMI) myocardial infarction of other sites |
| 410.71 | Subendocardial infarction, initial episode of care | I214 | Non-ST elevation (NSTEMI) myocardial infarction |
| 410.71 | Subendocardial infarction, initial episode of care | I222 | Subsequent non-ST elevation (NSTEMI) myocardial infarction |
| 410.81 | Acute myocardial infarction of other specified sites, initial episode of care | I2121 | ST elevation (STEMI) myocardial infarction involving left circumflex coronary artery |
| 410.81 | Acute myocardial infarction of other specified sites, initial episode of care | I2129 | ST elevation (STEMI) myocardial infarction involving other sites |
| 410.81 | Acute myocardial infarction of other specified sites, initial episode of care | I228 | Subsequent ST elevation (STEMI) myocardial infarction of other sites |
| 410.91 | Acute myocardial infarction of unspecified site, initial episode of care | I213 | ST elevation (STEMI) myocardial infarction of unspecified site |
| 410.91 | Acute myocardial infarction of unspecified site, initial episode of care | I229 | Subsequent ST elevation (STEMI) myocardial infarction of unspecified site |
| 411.1 | Intermediate coronary syndrome | I200 | Unstable angina |
| 411.1 | Intermediate coronary syndrome | I25110 | Atherosclerotic heart disease of native coronary artery with unstable angina pectoris |
| 411.1 | Intermediate coronary syndrome | I25700 | Atherosclerosis of coronary artery bypass graft(s), unspecified, with unstable angina pectoris |
| 411.1 | Intermediate coronary syndrome | I25710 | Atherosclerosis of autologous vein coronary artery bypass graft(s) with unstable angina pectoris |
| 411.1 | Intermediate coronary syndrome | I25720 | Atherosclerosis of autologous artery coronary artery bypass graft(s) with unstable angina pectoris |
| 411.1 | Intermediate coronary syndrome | I25730 | Atherosclerosis of nonautologous biological coronary artery bypass graft(s) with unstable angina pectoris |
| 411.1 | Intermediate coronary syndrome | I25750 | Atherosclerosis of native coronary artery of transplanted heart with unstable angina |
| 411.1 | Intermediate coronary syndrome | I25760 | Atherosclerosis of bypass graft of coronary artery of transplanted heart with unstable angina |
| 411.1 | Intermediate coronary syndrome | I25790 | Atherosclerosis of other coronary artery bypass graft(s) with unstable angina pectoris |
| 411.81 | Acute coronary occlusion without myocardial infarction | I240 | Acute coronary thrombosis not resulting in myocardial infarction |
| 411.89 | Other acute and subacute forms of ischemic heart disease, other | I248 | Other forms of acute ischemic heart disease |
| 411.89 | Other acute and subacute forms of ischemic heart disease, other | I249 | Acute ischemic heart disease, unspecified |
| 412. | Old myocardial infarction | I252 | Old myocardial infarction |
| 413.0 | Angina decubitus | I208 | Other forms of angina pectoris |
| 413.9 | Other and unspecified angina pectoris | I208 | Other forms of angina pectoris |
| 413.9 | Other and unspecified angina pectoris | I209 | Angina pectoris, unspecified |
| 413.9 | Other and unspecified angina pectoris | I25111 | Atherosclerotic heart disease of native coronary artery with angina pectoris with documented spasm |
| 413.9 | Other and unspecified angina pectoris | I25118 | Atherosclerotic heart disease of native coronary artery with other forms of angina pectoris |
| 413.9 | Other and unspecified angina pectoris | I25119 | Atherosclerotic heart disease of native coronary artery with unspecified angina pectoris |
| 413.9 | Other and unspecified angina pectoris | I25701 | Atherosclerosis of coronary artery bypass graft(s), unspecified, with angina pectoris with documented spasm |
| 413.9 | Other and unspecified angina pectoris | I25708 | Atherosclerosis of coronary artery bypass graft(s), unspecified, with other forms of angina pectoris |
| 413.9 | Other and unspecified angina pectoris | I25709 | Atherosclerosis of coronary artery bypass graft(s), unspecified, with unspecified angina pectoris |
| 413.9 | Other and unspecified angina pectoris | I25711 | Atherosclerosis of autologous vein coronary artery bypass graft(s) with angina pectoris with documented spasm |
| 413.9 | Other and unspecified angina pectoris | I25718 | Atherosclerosis of autologous vein coronary artery bypass graft(s) with other forms of angina pectoris |
| 413.9 | Other and unspecified angina pectoris | I25719 | Atherosclerosis of autologous vein coronary artery bypass graft(s) with unspecified angina pectoris |
| 413.9 | Other and unspecified angina pectoris | I25721 | Atherosclerosis of autologous artery coronary artery bypass graft(s) with angina pectoris with documented spasm |
| 413.9 | Other and unspecified angina pectoris | I25728 | Atherosclerosis of autologous artery coronary artery bypass graft(s) with other forms of angina pectoris |
| 413.9 | Other and unspecified angina pectoris | I25729 | Atherosclerosis of autologous artery coronary artery bypass graft(s) with unspecified angina pectoris |
| 413.9 | Other and unspecified angina pectoris | I25731 | Atherosclerosis of nonautologous biological coronary artery bypass graft(s) with angina pectoris with documented spasm |
| 413.9 | Other and unspecified angina pectoris | I25738 | Atherosclerosis of nonautologous biological coronary artery bypass graft(s) with other forms of angina pectoris |
| 413.9 | Other and unspecified angina pectoris | I25739 | Atherosclerosis of nonautologous biological coronary artery bypass graft(s) with unspecified angina pectoris |
| 413.9 | Other and unspecified angina pectoris | I25751 | Atherosclerosis of native coronary artery of transplanted heart with angina pectoris with documented spasm |
| 413.9 | Other and unspecified angina pectoris | I25758 | Atherosclerosis of native coronary artery of transplanted heart with other forms of angina pectoris |
| 413.9 | Other and unspecified angina pectoris | I25759 | Atherosclerosis of native coronary artery of transplanted heart with unspecified angina pectoris |
| 413.9 | Other and unspecified angina pectoris | I25761 | Atherosclerosis of bypass graft of coronary artery of transplanted heart with angina pectoris with documented spasm |
| 413.9 | Other and unspecified angina pectoris | I25768 | Atherosclerosis of bypass graft of coronary artery of transplanted heart with other forms of angina pectoris |
| 413.9 | Other and unspecified angina pectoris | I25769 | Atherosclerosis of bypass graft of coronary artery of transplanted heart with unspecified angina pectoris |
| 413.9 | Other and unspecified angina pectoris | I25791 | Atherosclerosis of other coronary artery bypass graft(s) with angina pectoris with documented spasm |
| 413.9 | Other and unspecified angina pectoris | I25798 | Atherosclerosis of other coronary artery bypass graft(s) with other forms of angina pectoris |
| 413.9 | Other and unspecified angina pectoris | I25799 | Atherosclerosis of other coronary artery bypass graft(s) with unspecified angina pectoris |
| 414.00 | Coronary atherosclerosis of unspecified type of vessel, native or graft | I2510 | Atherosclerotic heart disease of native coronary artery without angina pectoris |
| 414.01 | Coronary atherosclerosis of native coronary artery | I2510 | Atherosclerotic heart disease of native coronary artery without angina pectoris |
| 414.01 | Coronary atherosclerosis of native coronary artery | I25110 | Atherosclerotic heart disease of native coronary artery with unstable angina pectoris |
| 414.01 | Coronary atherosclerosis of native coronary artery | I25111 | Atherosclerotic heart disease of native coronary artery with angina pectoris with documented spasm |
| 414.01 | Coronary atherosclerosis of native coronary artery | I25118 | Atherosclerotic heart disease of native coronary artery with other forms of angina pectoris |
| 414.01 | Coronary atherosclerosis of native coronary artery | I25119 | Atherosclerotic heart disease of native coronary artery with unspecified angina pectoris |
| 414.02 | Coronary atherosclerosis of autologous vein bypass graft | I25710 | Atherosclerosis of autologous vein coronary artery bypass graft(s) with unstable angina pectoris |
| 414.02 | Coronary atherosclerosis of autologous vein bypass graft | I25711 | Atherosclerosis of autologous vein coronary artery bypass graft(s) with angina pectoris with documented spasm |
| 414.02 | Coronary atherosclerosis of autologous vein bypass graft | I25718 | Atherosclerosis of autologous vein coronary artery bypass graft(s) with other forms of angina pectoris |
| 414.02 | Coronary atherosclerosis of autologous vein bypass graft | I25719 | Atherosclerosis of autologous vein coronary artery bypass graft(s) with unspecified angina pectoris |
| 414.02 | Coronary atherosclerosis of autologous vein bypass graft | I25810 | Atherosclerosis of coronary artery bypass graft(s) without angina pectoris |
| 414.03 | Coronary atherosclerosis of nonautologous biological bypass graft | I25730 | Atherosclerosis of nonautologous biological coronary artery bypass graft(s) with unstable angina pectoris |
| 414.03 | Coronary atherosclerosis of nonautologous biological bypass graft | I25731 | Atherosclerosis of nonautologous biological coronary artery bypass graft(s) with angina pectoris with documented spasm |
| 414.03 | Coronary atherosclerosis of nonautologous biological bypass graft | I25738 | Atherosclerosis of nonautologous biological coronary artery bypass graft(s) with other forms of angina pectoris |
| 414.03 | Coronary atherosclerosis of nonautologous biological bypass graft | I25739 | Atherosclerosis of nonautologous biological coronary artery bypass graft(s) with unspecified angina pectoris |
| 414.03 | Coronary atherosclerosis of nonautologous biological bypass graft | I25810 | Atherosclerosis of coronary artery bypass graft(s) without angina pectoris |
| 414.04 | Coronary atherosclerosis of artery bypass graft | I25720 | Atherosclerosis of autologous artery coronary artery bypass graft(s) with unstable angina pectoris |
| 414.04 | Coronary atherosclerosis of artery bypass graft | I25721 | Atherosclerosis of autologous artery coronary artery bypass graft(s) with angina pectoris with documented spasm |
| 414.04 | Coronary atherosclerosis of artery bypass graft | I25728 | Atherosclerosis of autologous artery coronary artery bypass graft(s) with other forms of angina pectoris |
| 414.04 | Coronary atherosclerosis of artery bypass graft | I25729 | Atherosclerosis of autologous artery coronary artery bypass graft(s) with unspecified angina pectoris |
| 414.04 | Coronary atherosclerosis of artery bypass graft | I25790 | Atherosclerosis of other coronary artery bypass graft(s) with unstable angina pectoris |
| 414.04 | Coronary atherosclerosis of artery bypass graft | I25791 | Atherosclerosis of other coronary artery bypass graft(s) with angina pectoris with documented spasm |
| 414.04 | Coronary atherosclerosis of artery bypass graft | I25798 | Atherosclerosis of other coronary artery bypass graft(s) with other forms of angina pectoris |
| 414.04 | Coronary atherosclerosis of artery bypass graft | I25799 | Atherosclerosis of other coronary artery bypass graft(s) with unspecified angina pectoris |
| 414.04 | Coronary atherosclerosis of artery bypass graft | I25810 | Atherosclerosis of coronary artery bypass graft(s) without angina pectoris |
| 414.05 | Coronary atherosclerosis of unspecified bypass graft | I25700 | Atherosclerosis of coronary artery bypass graft(s), unspecified, with unstable angina pectoris |
| 414.05 | Coronary atherosclerosis of unspecified bypass graft | I25701 | Atherosclerosis of coronary artery bypass graft(s), unspecified, with angina pectoris with documented spasm |
| 414.05 | Coronary atherosclerosis of unspecified bypass graft | I25708 | Atherosclerosis of coronary artery bypass graft(s), unspecified, with other forms of angina pectoris |
| 414.05 | Coronary atherosclerosis of unspecified bypass graft | I25709 | Atherosclerosis of coronary artery bypass graft(s), unspecified, with unspecified angina pectoris |
| 414.05 | Coronary atherosclerosis of unspecified bypass graft | I25790 | Atherosclerosis of other coronary artery bypass graft(s) with unstable angina pectoris |
| 414.05 | Coronary atherosclerosis of unspecified bypass graft | I25791 | Atherosclerosis of other coronary artery bypass graft(s) with angina pectoris with documented spasm |
| 414.05 | Coronary atherosclerosis of unspecified bypass graft | I25798 | Atherosclerosis of other coronary artery bypass graft(s) with other forms of angina pectoris |
| 414.05 | Coronary atherosclerosis of unspecified bypass graft | I25799 | Atherosclerosis of other coronary artery bypass graft(s) with unspecified angina pectoris |
| 414.05 | Coronary atherosclerosis of unspecified bypass graft | I25810 | Atherosclerosis of coronary artery bypass graft(s) without angina pectoris |
| 414.06 | Coronary atherosclerosis of native coronary artery of transplanted heart | I25750 | Atherosclerosis of native coronary artery of transplanted heart with unstable angina |
| 414.06 | Coronary atherosclerosis of native coronary artery of transplanted heart | I25751 | Atherosclerosis of native coronary artery of transplanted heart with angina pectoris with documented spasm |
| 414.06 | Coronary atherosclerosis of native coronary artery of transplanted heart | I25758 | Atherosclerosis of native coronary artery of transplanted heart with other forms of angina pectoris |
| 414.06 | Coronary atherosclerosis of native coronary artery of transplanted heart | I25759 | Atherosclerosis of native coronary artery of transplanted heart with unspecified angina pectoris |
| 414.06 | Coronary atherosclerosis of native coronary artery of transplanted heart | I25811 | Atherosclerosis of native coronary artery of transplanted heart without angina pectoris |
| 414.07 | Coronary atherosclerosis of bypass graft (artery) (vein) of transplanted heart | I25760 | Atherosclerosis of bypass graft of coronary artery of transplanted heart with unstable angina |
| 414.07 | Coronary atherosclerosis of bypass graft (artery) (vein) of transplanted heart | I25761 | Atherosclerosis of bypass graft of coronary artery of transplanted heart with angina pectoris with documented spasm |
| 414.07 | Coronary atherosclerosis of bypass graft (artery) (vein) of transplanted heart | I25768 | Atherosclerosis of bypass graft of coronary artery of transplanted heart with other forms of angina pectoris |
| 414.07 | Coronary atherosclerosis of bypass graft (artery) (vein) of transplanted heart | I25769 | Atherosclerosis of bypass graft of coronary artery of transplanted heart with unspecified angina pectoris |
| 414.07 | Coronary atherosclerosis of bypass graft (artery) (vein) of transplanted heart | I25812 | Atherosclerosis of bypass graft of coronary artery of transplanted heart without angina pectoris |
| 414.2 | Chronic total occlusion of coronary artery | I2582 | Chronic total occlusion of coronary artery |
| 414.3 | Coronary atherosclerosis due to lipid rich plaque | I2583 | Coronary atherosclerosis due to lipid rich plaque |
| 414.4 | Coronary atherosclerosis due to calcified coronary lesion | I2584 | Coronary atherosclerosis due to calcified coronary lesion |
| 414.8 | Other specified forms of chronic ischemic heart disease | I255 | Ischemic cardiomyopathy |
| 414.8 | Other specified forms of chronic ischemic heart disease | I256 | Silent myocardial ischemia |
| 414.8 | Other specified forms of chronic ischemic heart disease | I2589 | Other forms of chronic ischemic heart disease |
| 414.8 | Other specified forms of chronic ischemic heart disease | I259 | Chronic ischemic heart disease, unspecified |
| 414.9 | Chronic ischemic heart disease, unspecified | I259 | Chronic ischemic heart disease, unspecified |
| 433.00 | Occlusion and stenosis of basilar artery without mention of cerebral infarction | I651 | Occlusion and stenosis of basilar artery |
| 433.01 | Occlusion and stenosis of basilar artery with cerebral infarction | I6302 | Cerebral infarction due to thrombosis of basilar artery |
| 433.01 | Occlusion and stenosis of basilar artery with cerebral infarction | I6312 | Cerebral infarction due to embolism of basilar artery |
| 433.01 | Occlusion and stenosis of basilar artery with cerebral infarction | I6322 | Cerebral infarction due to unspecified occlusion or stenosis of basilar arteries |
| 433.10 | Occlusion and stenosis of carotid artery without mention of cerebral infarction | I6521 | Occlusion and stenosis of right carotid artery |
| 433.10 | Occlusion and stenosis of carotid artery without mention of cerebral infarction | I6522 | Occlusion and stenosis of left carotid artery |
| 433.10 | Occlusion and stenosis of carotid artery without mention of cerebral infarction | I6523 | Occlusion and stenosis of bilateral carotid arteries |
| 433.10 | Occlusion and stenosis of carotid artery without mention of cerebral infarction | I6529 | Occlusion and stenosis of unspecified carotid artery |
| 433.11 | Occlusion and stenosis of carotid artery with cerebral infarction | I63031 | Cerebral infarction due to thrombosis of right carotid artery |
| 433.11 | Occlusion and stenosis of carotid artery with cerebral infarction | I63032 | Cerebral infarction due to thrombosis of left carotid artery |
| 433.11 | Occlusion and stenosis of carotid artery with cerebral infarction | I63039 | Cerebral infarction due to thrombosis of unspecified carotid artery |
| 433.11 | Occlusion and stenosis of carotid artery with cerebral infarction | I63131 | Cerebral infarction due to embolism of right carotid artery |
| 433.11 | Occlusion and stenosis of carotid artery with cerebral infarction | I63132 | Cerebral infarction due to embolism of left carotid artery |
| 433.11 | Occlusion and stenosis of carotid artery with cerebral infarction | I63139 | Cerebral infarction due to embolism of unspecified carotid artery |
| 433.11 | Occlusion and stenosis of carotid artery with cerebral infarction | I63231 | Cerebral infarction due to unspecified occlusion or stenosis of right carotid arteries |
| 433.11 | Occlusion and stenosis of carotid artery with cerebral infarction | I63232 | Cerebral infarction due to unspecified occlusion or stenosis of left carotid arteries |
| 433.11 | Occlusion and stenosis of carotid artery with cerebral infarction | I63239 | Cerebral infarction due to unspecified occlusion or stenosis of unspecified carotid arteries |
| 433.20 | Occlusion and stenosis of vertebral artery without mention of cerebral infarction | I6501 | Occlusion and stenosis of right vertebral artery |
| 433.20 | Occlusion and stenosis of vertebral artery without mention of cerebral infarction | I6502 | Occlusion and stenosis of left vertebral artery |
| 433.20 | Occlusion and stenosis of vertebral artery without mention of cerebral infarction | I6503 | Occlusion and stenosis of bilateral vertebral arteries |
| 433.20 | Occlusion and stenosis of vertebral artery without mention of cerebral infarction | I6509 | Occlusion and stenosis of unspecified vertebral artery |
| 433.21 | Occlusion and stenosis of vertebral artery with cerebral infarction | I63011 | Cerebral infarction due to thrombosis of right vertebral artery |
| 433.21 | Occlusion and stenosis of vertebral artery with cerebral infarction | I63012 | Cerebral infarction due to thrombosis of left vertebral artery |
| 433.21 | Occlusion and stenosis of vertebral artery with cerebral infarction | I63019 | Cerebral infarction due to thrombosis of unspecified vertebral artery |
| 433.21 | Occlusion and stenosis of vertebral artery with cerebral infarction | I63111 | Cerebral infarction due to embolism of right vertebral artery |
| 433.21 | Occlusion and stenosis of vertebral artery with cerebral infarction | I63112 | Cerebral infarction due to embolism of left vertebral artery |
| 433.21 | Occlusion and stenosis of vertebral artery with cerebral infarction | I63119 | Cerebral infarction due to embolism of unspecified vertebral artery |
| 433.21 | Occlusion and stenosis of vertebral artery with cerebral infarction | I63211 | Cerebral infarction due to unspecified occlusion or stenosis of right vertebral arteries |
| 433.21 | Occlusion and stenosis of vertebral artery with cerebral infarction | I63212 | Cerebral infarction due to unspecified occlusion or stenosis of left vertebral arteries |
| 433.21 | Occlusion and stenosis of vertebral artery with cerebral infarction | I63219 | Cerebral infarction due to unspecified occlusion or stenosis of unspecified vertebral arteries |
| 433.30 | Occlusion and stenosis of multiple and bilateral precerebral arteries without mention of cerebral infarction | I658 | Occlusion and stenosis of other precerebral arteries |
| 433.31 | Occlusion and stenosis of multiple and bilateral precerebral arteries with cerebral infarction | I6359 | Cerebral infarction due to unspecified occlusion or stenosis of other cerebral artery |
| 433.80 | Occlusion and stenosis of other specified precerebral artery without mention of cerebral infarction | I658 | Occlusion and stenosis of other precerebral arteries |
| 433.81 | Occlusion and stenosis of other specified precerebral artery with cerebral infarction | I6309 | Cerebral infarction due to thrombosis of other precerebral artery |
| 433.81 | Occlusion and stenosis of other specified precerebral artery with cerebral infarction | I6319 | Cerebral infarction due to embolism of other precerebral artery |
| 433.81 | Occlusion and stenosis of other specified precerebral artery with cerebral infarction | I6359 | Cerebral infarction due to unspecified occlusion or stenosis of other cerebral artery |
| 433.90 | Occlusion and stenosis of unspecified precerebral artery without mention of cerebral infarction | I659 | Occlusion and stenosis of unspecified precerebral artery |
| 433.91 | Occlusion and stenosis of unspecified precerebral artery with cerebral infarction | I6300 | Cerebral infarction due to thrombosis of unspecified precerebral artery |
| 433.91 | Occlusion and stenosis of unspecified precerebral artery with cerebral infarction | I6310 | Cerebral infarction due to embolism of unspecified precerebral artery |
| 433.91 | Occlusion and stenosis of unspecified precerebral artery with cerebral infarction | I6320 | Cerebral infarction due to unspecified occlusion or stenosis of unspecified precerebral arteries |
| 433.91 | Occlusion and stenosis of unspecified precerebral artery with cerebral infarction | I6329 | Cerebral infarction due to unspecified occlusion or stenosis of other precerebral arteries |
| 440.1 | Atherosclerosis of renal artery | I701 | Atherosclerosis of renal artery |
| 440.20 | Atherosclerosis of native arteries of the extremities, unspecified | I70201 | Unspecified atherosclerosis of native arteries of extremities, right leg |
| 440.20 | Atherosclerosis of native arteries of the extremities, unspecified | I70202 | Unspecified atherosclerosis of native arteries of extremities, left leg |
| 440.20 | Atherosclerosis of native arteries of the extremities, unspecified | I70203 | Unspecified atherosclerosis of native arteries of extremities, bilateral legs |
| 440.20 | Atherosclerosis of native arteries of the extremities, unspecified | I70208 | Unspecified atherosclerosis of native arteries of extremities, other extremity |
| 440.20 | Atherosclerosis of native arteries of the extremities, unspecified | I70209 | Unspecified atherosclerosis of native arteries of extremities, unspecified extremity |
| 440.21 | Atherosclerosis of native arteries of the extremities with intermittent claudication | I70211 | Atherosclerosis of native arteries of extremities with intermittent claudication, right leg |
| 440.21 | Atherosclerosis of native arteries of the extremities with intermittent claudication | I70212 | Atherosclerosis of native arteries of extremities with intermittent claudication, left leg |
| 440.21 | Atherosclerosis of native arteries of the extremities with intermittent claudication | I70213 | Atherosclerosis of native arteries of extremities with intermittent claudication, bilateral legs |
| 440.21 | Atherosclerosis of native arteries of the extremities with intermittent claudication | I70218 | Atherosclerosis of native arteries of extremities with intermittent claudication, other extremity |
| 440.21 | Atherosclerosis of native arteries of the extremities with intermittent claudication | I70219 | Atherosclerosis of native arteries of extremities with intermittent claudication, unspecified extremity |
| 440.22 | Atherosclerosis of native arteries of the extremities with rest pain | I70221 | Atherosclerosis of native arteries of extremities with rest pain, right leg |
| 440.22 | Atherosclerosis of native arteries of the extremities with rest pain | I70222 | Atherosclerosis of native arteries of extremities with rest pain, left leg |
| 440.22 | Atherosclerosis of native arteries of the extremities with rest pain | I70223 | Atherosclerosis of native arteries of extremities with rest pain, bilateral legs |
| 440.22 | Atherosclerosis of native arteries of the extremities with rest pain | I70228 | Atherosclerosis of native arteries of extremities with rest pain, other extremity |
| 440.22 | Atherosclerosis of native arteries of the extremities with rest pain | I70229 | Atherosclerosis of native arteries of extremities with rest pain, unspecified extremity |
| 440.23 | Atherosclerosis of native arteries of the extremities with ulceration | I70231 | Atherosclerosis of native arteries of right leg with ulceration of thigh |
| 440.23 | Atherosclerosis of native arteries of the extremities with ulceration | I70232 | Atherosclerosis of native arteries of right leg with ulceration of calf |
| 440.23 | Atherosclerosis of native arteries of the extremities with ulceration | I70233 | Atherosclerosis of native arteries of right leg with ulceration of ankle |
| 440.23 | Atherosclerosis of native arteries of the extremities with ulceration | I70234 | Atherosclerosis of native arteries of right leg with ulceration of heel and midfoot |
| 440.23 | Atherosclerosis of native arteries of the extremities with ulceration | I70235 | Atherosclerosis of native arteries of right leg with ulceration of other part of foot |
| 440.23 | Atherosclerosis of native arteries of the extremities with ulceration | I70238 | Atherosclerosis of native arteries of right leg with ulceration of other part of lower right leg |
| 440.23 | Atherosclerosis of native arteries of the extremities with ulceration | I70239 | Atherosclerosis of native arteries of right leg with ulceration of unspecified site |
| 440.23 | Atherosclerosis of native arteries of the extremities with ulceration | I70241 | Atherosclerosis of native arteries of left leg with ulceration of thigh |
| 440.23 | Atherosclerosis of native arteries of the extremities with ulceration | I70242 | Atherosclerosis of native arteries of left leg with ulceration of calf |
| 440.23 | Atherosclerosis of native arteries of the extremities with ulceration | I70243 | Atherosclerosis of native arteries of left leg with ulceration of ankle |
| 440.23 | Atherosclerosis of native arteries of the extremities with ulceration | I70244 | Atherosclerosis of native arteries of left leg with ulceration of heel and midfoot |
| 440.23 | Atherosclerosis of native arteries of the extremities with ulceration | I70245 | Atherosclerosis of native arteries of left leg with ulceration of other part of foot |
| 440.23 | Atherosclerosis of native arteries of the extremities with ulceration | I70248 | Atherosclerosis of native arteries of left leg with ulceration of other part of lower left leg |
| 440.23 | Atherosclerosis of native arteries of the extremities with ulceration | I70249 | Atherosclerosis of native arteries of left leg with ulceration of unspecified site |
| 440.23 | Atherosclerosis of native arteries of the extremities with ulceration | I7025 | Atherosclerosis of native arteries of other extremities with ulceration |
| 440.24 | Atherosclerosis of native arteries of the extremities with gangrene | I70261 | Atherosclerosis of native arteries of extremities with gangrene, right leg |
| 440.24 | Atherosclerosis of native arteries of the extremities with gangrene | I70262 | Atherosclerosis of native arteries of extremities with gangrene, left leg |
| 440.24 | Atherosclerosis of native arteries of the extremities with gangrene | I70263 | Atherosclerosis of native arteries of extremities with gangrene, bilateral legs |
| 440.24 | Atherosclerosis of native arteries of the extremities with gangrene | I70268 | Atherosclerosis of native arteries of extremities with gangrene, other extremity |
| 440.24 | Atherosclerosis of native arteries of the extremities with gangrene | I70269 | Atherosclerosis of native arteries of extremities with gangrene, unspecified extremity |
| 440.29 | Other atherosclerosis of native arteries of the extremities | I70291 | Other atherosclerosis of native arteries of extremities, right leg |
| 440.29 | Other atherosclerosis of native arteries of the extremities | I70292 | Other atherosclerosis of native arteries of extremities, left leg |
| 440.29 | Other atherosclerosis of native arteries of the extremities | I70293 | Other atherosclerosis of native arteries of extremities, bilateral legs |
| 440.29 | Other atherosclerosis of native arteries of the extremities | I70298 | Other atherosclerosis of native arteries of extremities, other extremity |
| 440.29 | Other atherosclerosis of native arteries of the extremities | I70299 | Other atherosclerosis of native arteries of extremities, unspecified extremity |
| 440.30 | Atherosclerosis of unspecified bypass graft of the extremities | I70301 | Unspecified atherosclerosis of unspecified type of bypass graft(s) of the extremities, right leg |
| 440.30 | Atherosclerosis of unspecified bypass graft of the extremities | I70302 | Unspecified atherosclerosis of unspecified type of bypass graft(s) of the extremities, left leg |
| 440.30 | Atherosclerosis of unspecified bypass graft of the extremities | I70303 | Unspecified atherosclerosis of unspecified type of bypass graft(s) of the extremities, bilateral legs |
| 440.30 | Atherosclerosis of unspecified bypass graft of the extremities | I70308 | Unspecified atherosclerosis of unspecified type of bypass graft(s) of the extremities, other extremity |
| 440.30 | Atherosclerosis of unspecified bypass graft of the extremities | I70309 | Unspecified atherosclerosis of unspecified type of bypass graft(s) of the extremities, unspecified extremity |
| 440.30 | Atherosclerosis of unspecified bypass graft of the extremities | I70311 | Atherosclerosis of unspecified type of bypass graft(s) of the extremities with intermittent claudication, right leg |
| 440.30 | Atherosclerosis of unspecified bypass graft of the extremities | I70312 | Atherosclerosis of unspecified type of bypass graft(s) of the extremities with intermittent claudication, left leg |
| 440.30 | Atherosclerosis of unspecified bypass graft of the extremities | I70313 | Atherosclerosis of unspecified type of bypass graft(s) of the extremities with intermittent claudication, bilateral legs |
| 440.30 | Atherosclerosis of unspecified bypass graft of the extremities | I70318 | Atherosclerosis of unspecified type of bypass graft(s) of the extremities with intermittent claudication, other extremity |
| 440.30 | Atherosclerosis of unspecified bypass graft of the extremities | I70319 | Atherosclerosis of unspecified type of bypass graft(s) of the extremities with intermittent claudication, unspecified extremity |
| 440.30 | Atherosclerosis of unspecified bypass graft of the extremities | I70321 | Atherosclerosis of unspecified type of bypass graft(s) of the extremities with rest pain, right leg |
| 440.30 | Atherosclerosis of unspecified bypass graft of the extremities | I70322 | Atherosclerosis of unspecified type of bypass graft(s) of the extremities with rest pain, left leg |
| 440.30 | Atherosclerosis of unspecified bypass graft of the extremities | I70323 | Atherosclerosis of unspecified type of bypass graft(s) of the extremities with rest pain, bilateral legs |
| 440.30 | Atherosclerosis of unspecified bypass graft of the extremities | I70328 | Atherosclerosis of unspecified type of bypass graft(s) of the extremities with rest pain, other extremity |
| 440.30 | Atherosclerosis of unspecified bypass graft of the extremities | I70329 | Atherosclerosis of unspecified type of bypass graft(s) of the extremities with rest pain, unspecified extremity |
| 440.30 | Atherosclerosis of unspecified bypass graft of the extremities | I70331 | Atherosclerosis of unspecified type of bypass graft(s) of the right leg with ulceration of thigh |
| 440.30 | Atherosclerosis of unspecified bypass graft of the extremities | I70332 | Atherosclerosis of unspecified type of bypass graft(s) of the right leg with ulceration of calf |
| 440.30 | Atherosclerosis of unspecified bypass graft of the extremities | I70333 | Atherosclerosis of unspecified type of bypass graft(s) of the right leg with ulceration of ankle |
| 440.30 | Atherosclerosis of unspecified bypass graft of the extremities | I70334 | Atherosclerosis of unspecified type of bypass graft(s) of the right leg with ulceration of heel and midfoot |
| 440.30 | Atherosclerosis of unspecified bypass graft of the extremities | I70335 | Atherosclerosis of unspecified type of bypass graft(s) of the right leg with ulceration of other part of foot |
| 440.30 | Atherosclerosis of unspecified bypass graft of the extremities | I70338 | Atherosclerosis of unspecified type of bypass graft(s) of the right leg with ulceration of other part of lower leg |
| 440.30 | Atherosclerosis of unspecified bypass graft of the extremities | I70339 | Atherosclerosis of unspecified type of bypass graft(s) of the right leg with ulceration of unspecified site |
| 440.30 | Atherosclerosis of unspecified bypass graft of the extremities | I70341 | Atherosclerosis of unspecified type of bypass graft(s) of the left leg with ulceration of thigh |
| 440.30 | Atherosclerosis of unspecified bypass graft of the extremities | I70342 | Atherosclerosis of unspecified type of bypass graft(s) of the left leg with ulceration of calf |
| 440.30 | Atherosclerosis of unspecified bypass graft of the extremities | I70343 | Atherosclerosis of unspecified type of bypass graft(s) of the left leg with ulceration of ankle |
| 440.30 | Atherosclerosis of unspecified bypass graft of the extremities | I70344 | Atherosclerosis of unspecified type of bypass graft(s) of the left leg with ulceration of heel and midfoot |
| 440.30 | Atherosclerosis of unspecified bypass graft of the extremities | I70345 | Atherosclerosis of unspecified type of bypass graft(s) of the left leg with ulceration of other part of foot |
| 440.30 | Atherosclerosis of unspecified bypass graft of the extremities | I70348 | Atherosclerosis of unspecified type of bypass graft(s) of the left leg with ulceration of other part of lower leg |
| 440.30 | Atherosclerosis of unspecified bypass graft of the extremities | I70349 | Atherosclerosis of unspecified type of bypass graft(s) of the left leg with ulceration of unspecified site |
| 440.30 | Atherosclerosis of unspecified bypass graft of the extremities | I7035 | Atherosclerosis of unspecified type of bypass graft(s) of other extremity with ulceration |
| 440.30 | Atherosclerosis of unspecified bypass graft of the extremities | I70361 | Atherosclerosis of unspecified type of bypass graft(s) of the extremities with gangrene, right leg |
| 440.30 | Atherosclerosis of unspecified bypass graft of the extremities | I70362 | Atherosclerosis of unspecified type of bypass graft(s) of the extremities with gangrene, left leg |
| 440.30 | Atherosclerosis of unspecified bypass graft of the extremities | I70363 | Atherosclerosis of unspecified type of bypass graft(s) of the extremities with gangrene, bilateral legs |
| 440.30 | Atherosclerosis of unspecified bypass graft of the extremities | I70368 | Atherosclerosis of unspecified type of bypass graft(s) of the extremities with gangrene, other extremity |
| 440.30 | Atherosclerosis of unspecified bypass graft of the extremities | I70369 | Atherosclerosis of unspecified type of bypass graft(s) of the extremities with gangrene, unspecified extremity |
| 440.30 | Atherosclerosis of unspecified bypass graft of the extremities | I70391 | Other atherosclerosis of unspecified type of bypass graft(s) of the extremities, right leg |
| 440.30 | Atherosclerosis of unspecified bypass graft of the extremities | I70392 | Other atherosclerosis of unspecified type of bypass graft(s) of the extremities, left leg |
| 440.30 | Atherosclerosis of unspecified bypass graft of the extremities | I70393 | Other atherosclerosis of unspecified type of bypass graft(s) of the extremities, bilateral legs |
| 440.30 | Atherosclerosis of unspecified bypass graft of the extremities | I70398 | Other atherosclerosis of unspecified type of bypass graft(s) of the extremities, other extremity |
| 440.30 | Atherosclerosis of unspecified bypass graft of the extremities | I70399 | Other atherosclerosis of unspecified type of bypass graft(s) of the extremities, unspecified extremity |
| 440.30 | Atherosclerosis of unspecified bypass graft of the extremities | I70601 | Unspecified atherosclerosis of nonbiological bypass graft(s) of the extremities, right leg |
| 440.30 | Atherosclerosis of unspecified bypass graft of the extremities | I70602 | Unspecified atherosclerosis of nonbiological bypass graft(s) of the extremities, left leg |
| 440.30 | Atherosclerosis of unspecified bypass graft of the extremities | I70603 | Unspecified atherosclerosis of nonbiological bypass graft(s) of the extremities, bilateral legs |
| 440.30 | Atherosclerosis of unspecified bypass graft of the extremities | I70608 | Unspecified atherosclerosis of nonbiological bypass graft(s) of the extremities, other extremity |
| 440.30 | Atherosclerosis of unspecified bypass graft of the extremities | I70609 | Unspecified atherosclerosis of nonbiological bypass graft(s) of the extremities, unspecified extremity |
| 440.30 | Atherosclerosis of unspecified bypass graft of the extremities | I70611 | Atherosclerosis of nonbiological bypass graft(s) of the extremities with intermittent claudication, right leg |
| 440.30 | Atherosclerosis of unspecified bypass graft of the extremities | I70612 | Atherosclerosis of nonbiological bypass graft(s) of the extremities with intermittent claudication, left leg |
| 440.30 | Atherosclerosis of unspecified bypass graft of the extremities | I70613 | Atherosclerosis of nonbiological bypass graft(s) of the extremities with intermittent claudication, bilateral legs |
| 440.30 | Atherosclerosis of unspecified bypass graft of the extremities | I70618 | Atherosclerosis of nonbiological bypass graft(s) of the extremities with intermittent claudication, other extremity |
| 440.30 | Atherosclerosis of unspecified bypass graft of the extremities | I70619 | Atherosclerosis of nonbiological bypass graft(s) of the extremities with intermittent claudication, unspecified extremity |
| 440.30 | Atherosclerosis of unspecified bypass graft of the extremities | I70621 | Atherosclerosis of nonbiological bypass graft(s) of the extremities with rest pain, right leg |
| 440.30 | Atherosclerosis of unspecified bypass graft of the extremities | I70622 | Atherosclerosis of nonbiological bypass graft(s) of the extremities with rest pain, left leg |
| 440.30 | Atherosclerosis of unspecified bypass graft of the extremities | I70623 | Atherosclerosis of nonbiological bypass graft(s) of the extremities with rest pain, bilateral legs |
| 440.30 | Atherosclerosis of unspecified bypass graft of the extremities | I70628 | Atherosclerosis of nonbiological bypass graft(s) of the extremities with rest pain, other extremity |
| 440.30 | Atherosclerosis of unspecified bypass graft of the extremities | I70629 | Atherosclerosis of nonbiological bypass graft(s) of the extremities with rest pain, unspecified extremity |
| 440.30 | Atherosclerosis of unspecified bypass graft of the extremities | I70631 | Atherosclerosis of nonbiological bypass graft(s) of the right leg with ulceration of thigh |
| 440.30 | Atherosclerosis of unspecified bypass graft of the extremities | I70632 | Atherosclerosis of nonbiological bypass graft(s) of the right leg with ulceration of calf |
| 440.30 | Atherosclerosis of unspecified bypass graft of the extremities | I70633 | Atherosclerosis of nonbiological bypass graft(s) of the right leg with ulceration of ankle |
| 440.30 | Atherosclerosis of unspecified bypass graft of the extremities | I70634 | Atherosclerosis of nonbiological bypass graft(s) of the right leg with ulceration of heel and midfoot |
| 440.30 | Atherosclerosis of unspecified bypass graft of the extremities | I70635 | Atherosclerosis of nonbiological bypass graft(s) of the right leg with ulceration of other part of foot |
| 440.30 | Atherosclerosis of unspecified bypass graft of the extremities | I70638 | Atherosclerosis of nonbiological bypass graft(s) of the right leg with ulceration of other part of lower leg |
| 440.30 | Atherosclerosis of unspecified bypass graft of the extremities | I70639 | Atherosclerosis of nonbiological bypass graft(s) of the right leg with ulceration of unspecified site |
| 440.30 | Atherosclerosis of unspecified bypass graft of the extremities | I70641 | Atherosclerosis of nonbiological bypass graft(s) of the left leg with ulceration of thigh |
| 440.30 | Atherosclerosis of unspecified bypass graft of the extremities | I70642 | Atherosclerosis of nonbiological bypass graft(s) of the left leg with ulceration of calf |
| 440.30 | Atherosclerosis of unspecified bypass graft of the extremities | I70643 | Atherosclerosis of nonbiological bypass graft(s) of the left leg with ulceration of ankle |
| 440.30 | Atherosclerosis of unspecified bypass graft of the extremities | I70644 | Atherosclerosis of nonbiological bypass graft(s) of the left leg with ulceration of heel and midfoot |
| 440.30 | Atherosclerosis of unspecified bypass graft of the extremities | I70645 | Atherosclerosis of nonbiological bypass graft(s) of the left leg with ulceration of other part of foot |
| 440.30 | Atherosclerosis of unspecified bypass graft of the extremities | I70648 | Atherosclerosis of nonbiological bypass graft(s) of the left leg with ulceration of other part of lower leg |
| 440.30 | Atherosclerosis of unspecified bypass graft of the extremities | I70649 | Atherosclerosis of nonbiological bypass graft(s) of the left leg with ulceration of unspecified site |
| 440.30 | Atherosclerosis of unspecified bypass graft of the extremities | I7065 | Atherosclerosis of nonbiological bypass graft(s) of other extremity with ulceration |
| 440.30 | Atherosclerosis of unspecified bypass graft of the extremities | I70661 | Atherosclerosis of nonbiological bypass graft(s) of the extremities with gangrene, right leg |
| 440.30 | Atherosclerosis of unspecified bypass graft of the extremities | I70662 | Atherosclerosis of nonbiological bypass graft(s) of the extremities with gangrene, left leg |
| 440.30 | Atherosclerosis of unspecified bypass graft of the extremities | I70663 | Atherosclerosis of nonbiological bypass graft(s) of the extremities with gangrene, bilateral legs |
| 440.30 | Atherosclerosis of unspecified bypass graft of the extremities | I70668 | Atherosclerosis of nonbiological bypass graft(s) of the extremities with gangrene, other extremity |
| 440.30 | Atherosclerosis of unspecified bypass graft of the extremities | I70669 | Atherosclerosis of nonbiological bypass graft(s) of the extremities with gangrene, unspecified extremity |
| 440.30 | Atherosclerosis of unspecified bypass graft of the extremities | I70691 | Other atherosclerosis of nonbiological bypass graft(s) of the extremities, right leg |
| 440.30 | Atherosclerosis of unspecified bypass graft of the extremities | I70692 | Other atherosclerosis of nonbiological bypass graft(s) of the extremities, left leg |
| 440.30 | Atherosclerosis of unspecified bypass graft of the extremities | I70693 | Other atherosclerosis of nonbiological bypass graft(s) of the extremities, bilateral legs |
| 440.30 | Atherosclerosis of unspecified bypass graft of the extremities | I70698 | Other atherosclerosis of nonbiological bypass graft(s) of the extremities, other extremity |
| 440.30 | Atherosclerosis of unspecified bypass graft of the extremities | I70699 | Other atherosclerosis of nonbiological bypass graft(s) of the extremities, unspecified extremity |
| 440.30 | Atherosclerosis of unspecified bypass graft of the extremities | I70701 | Unspecified atherosclerosis of other type of bypass graft(s) of the extremities, right leg |
| 440.30 | Atherosclerosis of unspecified bypass graft of the extremities | I70702 | Unspecified atherosclerosis of other type of bypass graft(s) of the extremities, left leg |
| 440.30 | Atherosclerosis of unspecified bypass graft of the extremities | I70703 | Unspecified atherosclerosis of other type of bypass graft(s) of the extremities, bilateral legs |
| 440.30 | Atherosclerosis of unspecified bypass graft of the extremities | I70708 | Unspecified atherosclerosis of other type of bypass graft(s) of the extremities, other extremity |
| 440.30 | Atherosclerosis of unspecified bypass graft of the extremities | I70709 | Unspecified atherosclerosis of other type of bypass graft(s) of the extremities, unspecified extremity |
| 440.30 | Atherosclerosis of unspecified bypass graft of the extremities | I70711 | Atherosclerosis of other type of bypass graft(s) of the extremities with intermittent claudication, right leg |
| 440.30 | Atherosclerosis of unspecified bypass graft of the extremities | I70712 | Atherosclerosis of other type of bypass graft(s) of the extremities with intermittent claudication, left leg |
| 440.30 | Atherosclerosis of unspecified bypass graft of the extremities | I70713 | Atherosclerosis of other type of bypass graft(s) of the extremities with intermittent claudication, bilateral legs |
| 440.30 | Atherosclerosis of unspecified bypass graft of the extremities | I70718 | Atherosclerosis of other type of bypass graft(s) of the extremities with intermittent claudication, other extremity |
| 440.30 | Atherosclerosis of unspecified bypass graft of the extremities | I70719 | Atherosclerosis of other type of bypass graft(s) of the extremities with intermittent claudication, unspecified extremity |
| 440.30 | Atherosclerosis of unspecified bypass graft of the extremities | I70721 | Atherosclerosis of other type of bypass graft(s) of the extremities with rest pain, right leg |
| 440.30 | Atherosclerosis of unspecified bypass graft of the extremities | I70722 | Atherosclerosis of other type of bypass graft(s) of the extremities with rest pain, left leg |
| 440.30 | Atherosclerosis of unspecified bypass graft of the extremities | I70723 | Atherosclerosis of other type of bypass graft(s) of the extremities with rest pain, bilateral legs |
| 440.30 | Atherosclerosis of unspecified bypass graft of the extremities | I70728 | Atherosclerosis of other type of bypass graft(s) of the extremities with rest pain, other extremity |
| 440.30 | Atherosclerosis of unspecified bypass graft of the extremities | I70729 | Atherosclerosis of other type of bypass graft(s) of the extremities with rest pain, unspecified extremity |
| 440.30 | Atherosclerosis of unspecified bypass graft of the extremities | I70731 | Atherosclerosis of other type of bypass graft(s) of the right leg with ulceration of thigh |
| 440.30 | Atherosclerosis of unspecified bypass graft of the extremities | I70732 | Atherosclerosis of other type of bypass graft(s) of the right leg with ulceration of calf |
| 440.30 | Atherosclerosis of unspecified bypass graft of the extremities | I70733 | Atherosclerosis of other type of bypass graft(s) of the right leg with ulceration of ankle |
| 440.30 | Atherosclerosis of unspecified bypass graft of the extremities | I70734 | Atherosclerosis of other type of bypass graft(s) of the right leg with ulceration of heel and midfoot |
| 440.30 | Atherosclerosis of unspecified bypass graft of the extremities | I70735 | Atherosclerosis of other type of bypass graft(s) of the right leg with ulceration of other part of foot |
| 440.30 | Atherosclerosis of unspecified bypass graft of the extremities | I70738 | Atherosclerosis of other type of bypass graft(s) of the right leg with ulceration of other part of lower leg |
| 440.30 | Atherosclerosis of unspecified bypass graft of the extremities | I70739 | Atherosclerosis of other type of bypass graft(s) of the right leg with ulceration of unspecified site |
| 440.30 | Atherosclerosis of unspecified bypass graft of the extremities | I70741 | Atherosclerosis of other type of bypass graft(s) of the left leg with ulceration of thigh |
| 440.30 | Atherosclerosis of unspecified bypass graft of the extremities | I70742 | Atherosclerosis of other type of bypass graft(s) of the left leg with ulceration of calf |
| 440.30 | Atherosclerosis of unspecified bypass graft of the extremities | I70743 | Atherosclerosis of other type of bypass graft(s) of the left leg with ulceration of ankle |
| 440.30 | Atherosclerosis of unspecified bypass graft of the extremities | I70744 | Atherosclerosis of other type of bypass graft(s) of the left leg with ulceration of heel and midfoot |
| 440.30 | Atherosclerosis of unspecified bypass graft of the extremities | I70745 | Atherosclerosis of other type of bypass graft(s) of the left leg with ulceration of other part of foot |
| 440.30 | Atherosclerosis of unspecified bypass graft of the extremities | I70748 | Atherosclerosis of other type of bypass graft(s) of the left leg with ulceration of other part of lower leg |
| 440.30 | Atherosclerosis of unspecified bypass graft of the extremities | I70749 | Atherosclerosis of other type of bypass graft(s) of the left leg with ulceration of unspecified site |
| 440.30 | Atherosclerosis of unspecified bypass graft of the extremities | I7075 | Atherosclerosis of other type of bypass graft(s) of other extremity with ulceration |
| 440.30 | Atherosclerosis of unspecified bypass graft of the extremities | I70761 | Atherosclerosis of other type of bypass graft(s) of the extremities with gangrene, right leg |
| 440.30 | Atherosclerosis of unspecified bypass graft of the extremities | I70762 | Atherosclerosis of other type of bypass graft(s) of the extremities with gangrene, left leg |
| 440.30 | Atherosclerosis of unspecified bypass graft of the extremities | I70763 | Atherosclerosis of other type of bypass graft(s) of the extremities with gangrene, bilateral legs |
| 440.30 | Atherosclerosis of unspecified bypass graft of the extremities | I70768 | Atherosclerosis of other type of bypass graft(s) of the extremities with gangrene, other extremity |
| 440.30 | Atherosclerosis of unspecified bypass graft of the extremities | I70769 | Atherosclerosis of other type of bypass graft(s) of the extremities with gangrene, unspecified extremity |
| 440.30 | Atherosclerosis of unspecified bypass graft of the extremities | I70791 | Other atherosclerosis of other type of bypass graft(s) of the extremities, right leg |
| 440.30 | Atherosclerosis of unspecified bypass graft of the extremities | I70792 | Other atherosclerosis of other type of bypass graft(s) of the extremities, left leg |
| 440.30 | Atherosclerosis of unspecified bypass graft of the extremities | I70793 | Other atherosclerosis of other type of bypass graft(s) of the extremities, bilateral legs |
| 440.30 | Atherosclerosis of unspecified bypass graft of the extremities | I70798 | Other atherosclerosis of other type of bypass graft(s) of the extremities, other extremity |
| 440.30 | Atherosclerosis of unspecified bypass graft of the extremities | I70799 | Other atherosclerosis of other type of bypass graft(s) of the extremities, unspecified extremity |
| 440.31 | Atherosclerosis of autologous vein bypass graft of the extremities | I70401 | Unspecified atherosclerosis of autologous vein bypass graft(s) of the extremities, right leg |
| 440.31 | Atherosclerosis of autologous vein bypass graft of the extremities | I70402 | Unspecified atherosclerosis of autologous vein bypass graft(s) of the extremities, left leg |
| 440.31 | Atherosclerosis of autologous vein bypass graft of the extremities | I70403 | Unspecified atherosclerosis of autologous vein bypass graft(s) of the extremities, bilateral legs |
| 440.31 | Atherosclerosis of autologous vein bypass graft of the extremities | I70408 | Unspecified atherosclerosis of autologous vein bypass graft(s) of the extremities, other extremity |
| 440.31 | Atherosclerosis of autologous vein bypass graft of the extremities | I70409 | Unspecified atherosclerosis of autologous vein bypass graft(s) of the extremities, unspecified extremity |
| 440.31 | Atherosclerosis of autologous vein bypass graft of the extremities | I70411 | Atherosclerosis of autologous vein bypass graft(s) of the extremities with intermittent claudication, right leg |
| 440.31 | Atherosclerosis of autologous vein bypass graft of the extremities | I70412 | Atherosclerosis of autologous vein bypass graft(s) of the extremities with intermittent claudication, left leg |
| 440.31 | Atherosclerosis of autologous vein bypass graft of the extremities | I70413 | Atherosclerosis of autologous vein bypass graft(s) of the extremities with intermittent claudication, bilateral legs |
| 440.31 | Atherosclerosis of autologous vein bypass graft of the extremities | I70418 | Atherosclerosis of autologous vein bypass graft(s) of the extremities with intermittent claudication, other extremity |
| 440.31 | Atherosclerosis of autologous vein bypass graft of the extremities | I70419 | Atherosclerosis of autologous vein bypass graft(s) of the extremities with intermittent claudication, unspecified extremity |
| 440.31 | Atherosclerosis of autologous vein bypass graft of the extremities | I70421 | Atherosclerosis of autologous vein bypass graft(s) of the extremities with rest pain, right leg |
| 440.31 | Atherosclerosis of autologous vein bypass graft of the extremities | I70422 | Atherosclerosis of autologous vein bypass graft(s) of the extremities with rest pain, left leg |
| 440.31 | Atherosclerosis of autologous vein bypass graft of the extremities | I70423 | Atherosclerosis of autologous vein bypass graft(s) of the extremities with rest pain, bilateral legs |
| 440.31 | Atherosclerosis of autologous vein bypass graft of the extremities | I70428 | Atherosclerosis of autologous vein bypass graft(s) of the extremities with rest pain, other extremity |
| 440.31 | Atherosclerosis of autologous vein bypass graft of the extremities | I70429 | Atherosclerosis of autologous vein bypass graft(s) of the extremities with rest pain, unspecified extremity |
| 440.31 | Atherosclerosis of autologous vein bypass graft of the extremities | I70431 | Atherosclerosis of autologous vein bypass graft(s) of the right leg with ulceration of thigh |
| 440.31 | Atherosclerosis of autologous vein bypass graft of the extremities | I70432 | Atherosclerosis of autologous vein bypass graft(s) of the right leg with ulceration of calf |
| 440.31 | Atherosclerosis of autologous vein bypass graft of the extremities | I70433 | Atherosclerosis of autologous vein bypass graft(s) of the right leg with ulceration of ankle |
| 440.31 | Atherosclerosis of autologous vein bypass graft of the extremities | I70434 | Atherosclerosis of autologous vein bypass graft(s) of the right leg with ulceration of heel and midfoot |
| 440.31 | Atherosclerosis of autologous vein bypass graft of the extremities | I70435 | Atherosclerosis of autologous vein bypass graft(s) of the right leg with ulceration of other part of foot |
| 440.31 | Atherosclerosis of autologous vein bypass graft of the extremities | I70438 | Atherosclerosis of autologous vein bypass graft(s) of the right leg with ulceration of other part of lower leg |
| 440.31 | Atherosclerosis of autologous vein bypass graft of the extremities | I70439 | Atherosclerosis of autologous vein bypass graft(s) of the right leg with ulceration of unspecified site |
| 440.31 | Atherosclerosis of autologous vein bypass graft of the extremities | I70441 | Atherosclerosis of autologous vein bypass graft(s) of the left leg with ulceration of thigh |
| 440.31 | Atherosclerosis of autologous vein bypass graft of the extremities | I70442 | Atherosclerosis of autologous vein bypass graft(s) of the left leg with ulceration of calf |
| 440.31 | Atherosclerosis of autologous vein bypass graft of the extremities | I70443 | Atherosclerosis of autologous vein bypass graft(s) of the left leg with ulceration of ankle |
| 440.31 | Atherosclerosis of autologous vein bypass graft of the extremities | I70444 | Atherosclerosis of autologous vein bypass graft(s) of the left leg with ulceration of heel and midfoot |
| 440.31 | Atherosclerosis of autologous vein bypass graft of the extremities | I70445 | Atherosclerosis of autologous vein bypass graft(s) of the left leg with ulceration of other part of foot |
| 440.31 | Atherosclerosis of autologous vein bypass graft of the extremities | I70448 | Atherosclerosis of autologous vein bypass graft(s) of the left leg with ulceration of other part of lower leg |
| 440.31 | Atherosclerosis of autologous vein bypass graft of the extremities | I70449 | Atherosclerosis of autologous vein bypass graft(s) of the left leg with ulceration of unspecified site |
| 440.31 | Atherosclerosis of autologous vein bypass graft of the extremities | I7045 | Atherosclerosis of autologous vein bypass graft(s) of other extremity with ulceration |
| 440.31 | Atherosclerosis of autologous vein bypass graft of the extremities | I70461 | Atherosclerosis of autologous vein bypass graft(s) of the extremities with gangrene, right leg |
| 440.31 | Atherosclerosis of autologous vein bypass graft of the extremities | I70462 | Atherosclerosis of autologous vein bypass graft(s) of the extremities with gangrene, left leg |
| 440.31 | Atherosclerosis of autologous vein bypass graft of the extremities | I70463 | Atherosclerosis of autologous vein bypass graft(s) of the extremities with gangrene, bilateral legs |
| 440.31 | Atherosclerosis of autologous vein bypass graft of the extremities | I70468 | Atherosclerosis of autologous vein bypass graft(s) of the extremities with gangrene, other extremity |
| 440.31 | Atherosclerosis of autologous vein bypass graft of the extremities | I70469 | Atherosclerosis of autologous vein bypass graft(s) of the extremities with gangrene, unspecified extremity |
| 440.31 | Atherosclerosis of autologous vein bypass graft of the extremities | I70491 | Other atherosclerosis of autologous vein bypass graft(s) of the extremities, right leg |
| 440.31 | Atherosclerosis of autologous vein bypass graft of the extremities | I70492 | Other atherosclerosis of autologous vein bypass graft(s) of the extremities, left leg |
| 440.31 | Atherosclerosis of autologous vein bypass graft of the extremities | I70493 | Other atherosclerosis of autologous vein bypass graft(s) of the extremities, bilateral legs |
| 440.31 | Atherosclerosis of autologous vein bypass graft of the extremities | I70498 | Other atherosclerosis of autologous vein bypass graft(s) of the extremities, other extremity |
| 440.31 | Atherosclerosis of autologous vein bypass graft of the extremities | I70499 | Other atherosclerosis of autologous vein bypass graft(s) of the extremities, unspecified extremity |
| 440.32 | Atherosclerosis of nonautologous biological bypass graft of the extremities | I70501 | Unspecified atherosclerosis of nonautologous biological bypass graft(s) of the extremities, right leg |
| 440.32 | Atherosclerosis of nonautologous biological bypass graft of the extremities | I70502 | Unspecified atherosclerosis of nonautologous biological bypass graft(s) of the extremities, left leg |
| 440.32 | Atherosclerosis of nonautologous biological bypass graft of the extremities | I70503 | Unspecified atherosclerosis of nonautologous biological bypass graft(s) of the extremities, bilateral legs |
| 440.32 | Atherosclerosis of nonautologous biological bypass graft of the extremities | I70508 | Unspecified atherosclerosis of nonautologous biological bypass graft(s) of the extremities, other extremity |
| 440.32 | Atherosclerosis of nonautologous biological bypass graft of the extremities | I70509 | Unspecified atherosclerosis of nonautologous biological bypass graft(s) of the extremities, unspecified extremity |
| 440.32 | Atherosclerosis of nonautologous biological bypass graft of the extremities | I70511 | Atherosclerosis of nonautologous biological bypass graft(s) of the extremities with intermittent claudication, right leg |
| 440.32 | Atherosclerosis of nonautologous biological bypass graft of the extremities | I70512 | Atherosclerosis of nonautologous biological bypass graft(s) of the extremities with intermittent claudication, left leg |
| 440.32 | Atherosclerosis of nonautologous biological bypass graft of the extremities | I70513 | Atherosclerosis of nonautologous biological bypass graft(s) of the extremities with intermittent claudication, bilateral legs |
| 440.32 | Atherosclerosis of nonautologous biological bypass graft of the extremities | I70518 | Atherosclerosis of nonautologous biological bypass graft(s) of the extremities with intermittent claudication, other extremity |
| 440.32 | Atherosclerosis of nonautologous biological bypass graft of the extremities | I70519 | Atherosclerosis of nonautologous biological bypass graft(s) of the extremities with intermittent claudication, unspecified extremity |
| 440.32 | Atherosclerosis of nonautologous biological bypass graft of the extremities | I70521 | Atherosclerosis of nonautologous biological bypass graft(s) of the extremities with rest pain, right leg |
| 440.32 | Atherosclerosis of nonautologous biological bypass graft of the extremities | I70522 | Atherosclerosis of nonautologous biological bypass graft(s) of the extremities with rest pain, left leg |
| 440.32 | Atherosclerosis of nonautologous biological bypass graft of the extremities | I70523 | Atherosclerosis of nonautologous biological bypass graft(s) of the extremities with rest pain, bilateral legs |
| 440.32 | Atherosclerosis of nonautologous biological bypass graft of the extremities | I70528 | Atherosclerosis of nonautologous biological bypass graft(s) of the extremities with rest pain, other extremity |
| 440.32 | Atherosclerosis of nonautologous biological bypass graft of the extremities | I70529 | Atherosclerosis of nonautologous biological bypass graft(s) of the extremities with rest pain, unspecified extremity |
| 440.32 | Atherosclerosis of nonautologous biological bypass graft of the extremities | I70531 | Atherosclerosis of nonautologous biological bypass graft(s) of the right leg with ulceration of thigh |
| 440.32 | Atherosclerosis of nonautologous biological bypass graft of the extremities | I70532 | Atherosclerosis of nonautologous biological bypass graft(s) of the right leg with ulceration of calf |
| 440.32 | Atherosclerosis of nonautologous biological bypass graft of the extremities | I70533 | Atherosclerosis of nonautologous biological bypass graft(s) of the right leg with ulceration of ankle |
| 440.32 | Atherosclerosis of nonautologous biological bypass graft of the extremities | I70534 | Atherosclerosis of nonautologous biological bypass graft(s) of the right leg with ulceration of heel and midfoot |
| 440.32 | Atherosclerosis of nonautologous biological bypass graft of the extremities | I70535 | Atherosclerosis of nonautologous biological bypass graft(s) of the right leg with ulceration of other part of foot |
| 440.32 | Atherosclerosis of nonautologous biological bypass graft of the extremities | I70538 | Atherosclerosis of nonautologous biological bypass graft(s) of the right leg with ulceration of other part of lower leg |
| 440.32 | Atherosclerosis of nonautologous biological bypass graft of the extremities | I70539 | Atherosclerosis of nonautologous biological bypass graft(s) of the right leg with ulceration of unspecified site |
| 440.32 | Atherosclerosis of nonautologous biological bypass graft of the extremities | I70541 | Atherosclerosis of nonautologous biological bypass graft(s) of the left leg with ulceration of thigh |
| 440.32 | Atherosclerosis of nonautologous biological bypass graft of the extremities | I70542 | Atherosclerosis of nonautologous biological bypass graft(s) of the left leg with ulceration of calf |
| 440.32 | Atherosclerosis of nonautologous biological bypass graft of the extremities | I70543 | Atherosclerosis of nonautologous biological bypass graft(s) of the left leg with ulceration of ankle |
| 440.32 | Atherosclerosis of nonautologous biological bypass graft of the extremities | I70544 | Atherosclerosis of nonautologous biological bypass graft(s) of the left leg with ulceration of heel and midfoot |
| 440.32 | Atherosclerosis of nonautologous biological bypass graft of the extremities | I70545 | Atherosclerosis of nonautologous biological bypass graft(s) of the left leg with ulceration of other part of foot |
| 440.32 | Atherosclerosis of nonautologous biological bypass graft of the extremities | I70548 | Atherosclerosis of nonautologous biological bypass graft(s) of the left leg with ulceration of other part of lower leg |
| 440.32 | Atherosclerosis of nonautologous biological bypass graft of the extremities | I70549 | Atherosclerosis of nonautologous biological bypass graft(s) of the left leg with ulceration of unspecified site |
| 440.32 | Atherosclerosis of nonautologous biological bypass graft of the extremities | I7055 | Atherosclerosis of nonautologous biological bypass graft(s) of other extremity with ulceration |
| 440.32 | Atherosclerosis of nonautologous biological bypass graft of the extremities | I70561 | Atherosclerosis of nonautologous biological bypass graft(s) of the extremities with gangrene, right leg |
| 440.32 | Atherosclerosis of nonautologous biological bypass graft of the extremities | I70562 | Atherosclerosis of nonautologous biological bypass graft(s) of the extremities with gangrene, left leg |
| 440.32 | Atherosclerosis of nonautologous biological bypass graft of the extremities | I70563 | Atherosclerosis of nonautologous biological bypass graft(s) of the extremities with gangrene, bilateral legs |
| 440.32 | Atherosclerosis of nonautologous biological bypass graft of the extremities | I70568 | Atherosclerosis of nonautologous biological bypass graft(s) of the extremities with gangrene, other extremity |
| 440.32 | Atherosclerosis of nonautologous biological bypass graft of the extremities | I70569 | Atherosclerosis of nonautologous biological bypass graft(s) of the extremities with gangrene, unspecified extremity |
| 440.32 | Atherosclerosis of nonautologous biological bypass graft of the extremities | I70591 | Other atherosclerosis of nonautologous biological bypass graft(s) of the extremities, right leg |
| 440.32 | Atherosclerosis of nonautologous biological bypass graft of the extremities | I70592 | Other atherosclerosis of nonautologous biological bypass graft(s) of the extremities, left leg |
| 440.32 | Atherosclerosis of nonautologous biological bypass graft of the extremities | I70593 | Other atherosclerosis of nonautologous biological bypass graft(s) of the extremities, bilateral legs |
| 440.32 | Atherosclerosis of nonautologous biological bypass graft of the extremities | I70598 | Other atherosclerosis of nonautologous biological bypass graft(s) of the extremities, other extremity |
| 440.32 | Atherosclerosis of nonautologous biological bypass graft of the extremities | I70599 | Other atherosclerosis of nonautologous biological bypass graft(s) of the extremities, unspecified extremity |
| 440.4 | Chronic total occlusion of artery of the extremities | I7092 | Chronic total occlusion of artery of the extremities |
